# An updated italian normative data for a short version of the stroop colour word test

**DOI:** 10.1007/s10072-026-08918-4

**Published:** 2026-03-25

**Authors:** Simona Luzzi, Veronica Cherubini, Pamela Rosettani, Sara Baldinelli, Chiara Fiori, Mauro Silvestrini, Michele Scandola

**Affiliations:** 1 Unit of Cognitive and Behavioural Neurology, Neurologic Unit, Department of Experimental and Clinical Medicine, Azienda Ospedaliero Universitaria delle Marche, Ancona, Italy; 2 Department of Human Sciences, University of Verona, Verona, Italy; 3 Cognitive and Behavioural Neurology Unit, Department of Experimental and Clinical Medicine, Neurologic Clinic, Polytechnic University of Marche, Ancona, Italy

**Keywords:** Attention, Stroop test, Neuropsychology, Dementia, Alzheimer’s disease

## Abstract

**Background:**

The Stroop Colour Word Test (SCWT) is a neuropsychological tool widely used to assess the ability to inhibit cognitive interference, particularly in patients with dementia. This study aimed to provide normative data for a short version of the SCWT in an Italian sample of 452 healthy individuals aged 20–90 years (education ≥ 5 years).

**Methods:**

The test version included 50 items using four colours (yellow, red, green, blue) and involved four tasks: (1) reading colour names in black ink, (2) naming coloured dots, (3) reading colour names, and (4) naming the ink colour of incongruent colour words. For each task, response time and errors were recorded. To analyze performance, we developed linear models including all combinations of the independent variables (sex, age, education), applying transformations (square root, logarithm, reciprocal, quadratic, cubic) to account for non-linear effects.

**Results:**

In total, 98 models per score were generated and assessed using the Bayesian Information Criterion (BIC). Correction factors were derived by comparing predicted scores from the best-fitting models to observed averages. The most accurate predictive models were identified for each performance index.

**Conclusions:**

The availability of these normative values enhances the clinical utility of the short SCWT in evaluating selective attention and interference control in both adults and older adults. This updated dataset contributes to refining neuropsychological assessments in this cognitive domain.

**Supplementary Information:**

The online version contains supplementary material available at 10.1007/s10072-026-08918-4.

## Introduction

The Stroop colour and word test (SCWT) [1] is a widely used test to evaluate selective attention, speed of information processing, response inhibition, working memory [2], sustained attention ad cognitive flexibility [3]. The classical version of the SCWT is composed of three tables, showing colour words, coloured squares or circles, and colour words printed in incongruent ink (i.e., red printed in blue ink), respectively.

The original test was developed by John Ridley Stroop in 1935 [1], and several versions are now in use. Although they differ in stimuli and task sequence [4], all demonstrate the Stroop effect—longer reaction times caused by interference when individuals must inhibit automatic reading in favor of naming ink colours in incongruent colour-word stimuli [1].

The SCWT evaluates the reaction times both to non-ambiguous stimuli (reading words in black ink or naming colours in painted forms) and to ambiguous stimuli (colour word printed with incongruent ink), thus assessing the inhibition mechanisms that are crucial to executive functions.

Although the WCST is commonly used to assess frontal lobe function, neuroanatomical evidence suggests it engages a broader network, including the dorsolateral prefrontal cortex, anterior cingulate cortex [5], posterior parietal cortex [6, 7], middle frontal gyrus, motor areas, and temporal regions [8]. The SCWT is widely applied clinically not only for suspected frontal lobe dysfunction but also in neurodegenerative diseases like Alzheimer’s Disease (AD) [9], Frontotemporal Dementia [10, 11], Lewy body Disease [11], Parkinson’s Disease [12], and vascular dementia [13]. It is also impaired in psychiatric conditions such as schizophrenia [14], depression [15], bipolar disorder [16], anorexia [17], and ADHD [18].

Several demographical factors are able to influence performance and in particular reaction times in the SCWT. The first one is aging that negatively affects the test performance [7, 19–21].

It is also reported that some activation patterns are slightly different between elderly and young people [7, 22].

Several studies also reported a significant effect of education on the SCWT performance. Subjects with higher education had shorter completion times [7, 20].

This study aimed to provide normative data for a short version of the SCWT in a sample of 452 healthy Italian participants, balanced by gender, age, and education. Although five Italian standardizations exist, they differ in number of items, tasks, scoring methods, and sample size. We used a version with 50 stimuli and four colour/words, with the addition of a Reverse Stroop task, here referred to as “Task 3”, in which participants are required to read colour words while ignoring the ink colour [23]. The Reverse Stroop effect has been described as the interference of ink colour on word reading [23, 24] and has traditionally been considered weak or absent in oral tasks, given the high automaticity of reading [23]. The rationale for including this task, instead of relying solely on the two traditional control tasks, is that Task 3 employs exactly the same stimuli as the interference condition, differing only in task demands. This choice allows for improved control of perceptual and lexical confounds, such that performance differences between Task 3 and Task 4 can be more directly attributed to automatic ink-colour processing, which characterizes Task 4. The number of 50 items was chosen for two main reasons.First, literature supports the use of short SCWT forms in clinical practice [19, 25]. Kang et al. [25] showed that a 50-item version captures the Stroop effect comparably to the full test, making it suitable for assessing processing speed and inhibition in older adults.

Second, based on our clinical experience, the 30-item version (e.g., Caffarra’s [26]) may lack sensitivity in early dementia stages, while the 100-item version [7, 27] is often too demanding for individuals over 65, who may lose focus midway [25].

Lastly, existing Italian norms are outdated—published 10 to 25 years ago [7, 26, 28]—and no longer reflect current demographic and cultural shifts, such as increased education and life expectancy, which influence cognitive performance in the general population.

## Methods

### Participants

An a-priori power analysis was executed to determine the minimum sample size to effectively study the effects of three regressors (i.e., age, education and sex) on the test’s raw scores. We used as effect size a Cohen’s f2 = 0.03 (a value between a small and medium effect size), with a power of the 80% and a standard alpha = 0.05, by means of the function from the pwr package [29] pwr.f2.test in R. The suggested sample size is 363 participants.

The study was performed on four hundred and fifty-two healthy participants (252 females and 200 males). All were Italian native speakers, with a mean age of 55.50 ± 17.04 years (males: 55.04 ± 17.25 years; females: 55.86 ± 16.89 years) and mean education of 10.58 ± 4.45 years (males: 10.84 ± 4.27 years; females: 10.37 ± 4.59 years). Moreover, we also collected a retrospective sample of 263 participants, divided into 124 with Alzheimer (MMSE = 18.8 ± 4.3; 78 females, age = 76.03 ± 6.39, education = 7.59 ± 4.65; 46 males, age = 74.80 ± 6.77, education = 9.06 ± 4.29) disease and 139 with MCI (MMSE = 25.9 ± 2.4; 68 females, age = 72.96 ± 7.81, education = 8.03 ± 3.84; 71 males, age = 71.59 ± 10.74, education = 9.87 ± 4.46) to validate the new norms (Table 1).

**Table 1:** Demographics. Table of the frequencies of participants divided by age and education groups. Sex frequencies are reported as m/f between round brackets. Edu = Years of education

Edu Age	0–4	5–8	9–13	14–19	Tot	Mean age (SD)	Mean Edu (SD)
20–24	-	-	-	14 (8/6)	14 (8/6)	22.143 (0.949)	14.857 (1.875)
25–29	-	-	6 (4/2)	16 (5/11)	22 (9/13)	27.091 (1.477)	13.364 (3.774)
30–34	-	-	8 (5/3)	15 (7/8)	23 (12/11)	31.739 (1.356)	12.174 (3.473)
35–39	-	-	11 (4/7)	18 (9/9)	29 (13/16)	36.793 (1.634)	12.345 (3.558)
40–44	1 (0/1)	4 (1/3)	9 (5/4)	26 (10/16)	40 (16/24)	41.875 (1.453)	11.85 (4.08)
45–49	-	5 (2/3)	14 (7/7)	16 (7/9)	35 (16/19)	46.943 (1.413)	10.8 (4.093)
50–54	-	10 (1/9)	11 (5/6)	32 (15/17)	53 (21/32)	51.962 (1.454)	11.302 (4.236)
55–59	1 (0/1)	14 (8/6)	13 (7/6)	19 (9/10)	47 (24/23)	57.128 (1.361)	10.128 (4.637)
60–64	1 (0/1)	10 (4/6)	12 (5/7)	14 (5/9)	37 (14/23)	61.892 (1.41)	9.784 (4.379)
65–69	3 (2/1)	14 (7/7)	10 (4/6)	11 (5/6)	38 (18/20)	66.816 (1.333)	8.763 (4.136)
70–74	4 (1/3)	11 (5/6)	12 (5/7)	19 (10/9)	46 (21/25)	71.957 (1.282)	9.848 (4.686)
75–79	4 (1/3)	13 (4/9)	4 (2/2)	10 (5/5)	31 (12/19)	77.129 (1.231)	8.323 (4.49)
80–84	3 (2/1)	8 (1/7)	6 (4/2)	6 (3/3)	23 (10/13)	81.696 (1.295)	8.043 (4.237)
85–90	3 (0/3)	4 (2/2)	2 (1/1)	5 (3/2)	14 (6/8)	86.786 (1.718)	8.857 (4.975)
Tot	20 (6/14)	93 (35/58)	118 (58/60)	221 (101/120)	452 (200/252)	55.496 (17.038)	10.58 (4.452)

The selection was made from among healthy subjects who came to the Cognitive and Behavioural Neurology Unit of the University Hospital of Marche. All subjects were chosen between spouses that were not part of the same genealogical tree of the patient.

Inclusion criteria were the following: absence of cognitive problems in their past clinical.

history, corrected scores for Mini-Mental State Examination [7] greater than 27 and corrected scores for Raven’s Coloured Progressive Matrices (R-CPM) greater than the normality cut-off score [7], any disease or condition that could alter cognitive performance such as: current or previous brain pathologies (e.g. stroke, head trauma, multiple sclerosis, degenerative brain diseases etc.); past or present history of addiction or alcohol abuse; major psychiatric disorders necessitating pharmacological intervention; systemic illnesses capable of altering the cognitive status such as severe diabetes with systemic complications, uncontrolled systemic hypertension (more than two drugs), renal or liver failure, untreated hyper/hypothyroidism; recent history of cancer and poly chemotherapy or radiotherapy; infancy or adolescent developmental disorders; sensory deficits (hypoacusia, visual problems). Neurological examination was performed in all subjects.

All participants provided informed consent for the utilisation of their data for research purposes, data acquisition was in accordance with the principles of the Helsinki Declaration, and the study was approved by the Local Ethic Committee (protocol number 2024/57).

### Procedure

Participants were asked to perform 4 tasks:

1. To read 50 words which are names of colour printed in black ink.
2. To name 50 colour patches (coloured rectangles).
3. To read 50 names of colour printed in an incongruent colour ink (e.g. the word “green” is printed with yellow ink).
4. To name the colour of the ink of incongruently coloured words.

Materials are reported in the supplementary materials and are composed by three white sheets A4 format; stimuli are located in the same spatial locations in each of the three sheets of paper. The third sheet of paper is used for tasks 3 and 4.

Tasks 1 and 2 are considered “congruous conditions”, while tasks 3 and 4 are considered “incongruous conditions”, because in those cases there is incongruence between the word and ink related.

For the sample of patients we only had data for task 4 and task 3.

### Data Handling

From the SCWT we extracted the following measures: (1) the time and the number of errors to read the colours names printed with black ink (T1 and E1, time and errors, respectively) (2) the time and errors to name the colours of dots (time = T2 and errors = E2); (3) the time and errors to read the colours names printed with incongruent colour (time = T3 and errors = E3); (4) the time and errors to name the colour of colour word printed with incongruent colour (time = T4 and errors = E4).

Three index were calculated: (a) the difference in time and error in reading tasks (3 − 1), i.e. the difference in time and number of errors between colour word written with black versus incongruent colour (T3-T1 and E3-E1) (b) the difference in naming tasks (4 − 2) i.e. the difference in time and number of errors between the coloured dots and the colour words (T4-T2 and E4-E2); (c) the time of resistance to interference and the number of error due to interference (T4-T3 and E4-E3).

### Statistical analyses

In order to confirm the different difficulties in terms of times and errors in the four SCWT tasks, a repeated-measures ANOVA, with Greenhouse–Geisser sphericity correction, was applied to the times of completion of each task, and a repeated-measures Generalised Linear model for binomial data for the errors. Post-hoc analyses were conducted on the estimated marginal means of the models, using the Holm-Bonferroni correction.

To determine the correction scores to be applied to the raw scores, we employed several linear models using as dependent variable one among T4, E4, T3, E3, T2, E2, T1 (we did not used E1 because the values were all constantly equal to zero) and the differences between T4 and T3, E4 and E3, T4 and T2, E4 and E2, T3 and T1 and E3 and E1 performance indices for the SCWT. When the differences were less than zero, they were recorded as zero. Subsequently, we constructed linear models encompassing all possible combinations of the independent variables—sex, age, and education—ranging from the null model (lacking any independent variable) to the saturated model (including all three independent variables). Additionally, age and education underwent various transformations: none, square root, quadratic, cubic, natural logarithm, reciprocal functions, and for age only ln(100 - age). All continuous independent variables were centred.

A total of 111 models were generated for each score, which were then evaluated using the Bayesian Information Criterion (BIC). The optimal predictive model was selected based on a criterion where its BIC was at least 2 points lower than other models, or if there were models within 2 points of difference, the model with fewer parameters was favoured. This approach adheres to a cutoff of 2 points, as it signifies favourable support towards the model with the lowest BIC [30]. Moreover, we computed the R2 as goodness of fit index, and we tested whether the independent variables were statistically significant after Bonferroni correction for multiple comparisons. This procedure is similar to the Arcara [31] and Gasparini and colleagues [32] procedures.

The adjusted data were computed applying the correction terms to the raw data, following the procedure proposed by Capitani & Laiacona [33]. Then, the adjusted data were standardised as Equivalent Scores (ES) [33]. The ES standardisation is typical in the Italian neuropsychological context. This approach allows to compute an outer tolerance limit (OTL), representing the cut-off guaranteeing that no more than the 5% of the reference population has a lower score, and an inner tolerance limit (ITL), guaranteeing that no less than 5% of the reference population has a score below it. Then, starting from the OTL, the cut-offs for the ESs are computed. ES is a 5-point scale, where ESs equal to 0 and 1 meaning defective and borderline, respectively; ES equals to 2 meaning low-end normal and ESs equal to 3 and 4 meaning normal. In this paper we followed the procedure suggested by Facchin and colleagues [34]. This approach follows a non-parametric procedure based on rank subdivision. Scores worse than the OTL are classified as ES = 0, scores better than the median are classified as ES = 4. The three intermediate scores are computed in order that the three portions of the distribution of the score have the same density [34].

Finally, for T4, E4, T3, E3, as well as the difference scores T4–T3 and E4–E3, corrected for age, education and sex as computed above, we conducted Receiver Operating Characteristic (ROC) analyses to evaluate the discriminative ability of these measures. ROC analyses were performed for the following group comparisons: patients with Alzheimer’s disease versus healthy participants, patients with mild cognitive impairment (MCI) versus healthy participants, and patients with Alzheimer’s disease versus MCI. For each ROC analysis, the optimal cut-off value was determined by maximizing the Youden index. Results are reported in terms of the optimal cut-off, Youden index, area under the curve (AUC), sensitivity, specificity, prevalence, accuracy, positive predictive value (PPV), and negative predictive value (NPV).All statistical analyses were conducted using R version 4.3.3 (R Core Team, 2024), and the packages tolerance version 2.0.0 [35], pwr for the a-priori power analysis [29] and cutpointr for the ROC analysis [36].

## Results

### Comparing the performances between the four tasks in the normative sample

Completion times differed significantly across the four tasks (F(1.40, 631.20) = 1681.80, *p* <.001, η² = 0.55), with all pairwise comparisons reaching significance after Holm–Bonferroni correction (all *p* <.001). However, the effect sizes between the first three tasks were small or negligible (Task 1 vs. Task 2: d = − 0.13; Task 1 vs. Task 3: d = − 0.09; Task 2 vs. Task 3: d = 0.04). In contrast, comparisons involving Task 4 consistently yielded large effect sizes (Task 1 vs. Task 4: d = − 0.76; Task 2 vs. Task 4: d = − 0.64; Task 3 vs. Task 4: d = − 0.67), indicating that Task 4 required substantially more time to complete.

Error analyses excluded Task 1, as errors were uniformly zero. A Generalized Linear Model revealed a significant effect of task (χ²(2) = 416.61, *p* <.001), driven by higher error rates in Task 4 compared with Tasks 2 and 3 (all Holm–Bonferroni corrected *p* <.001).

Descriptive statistics from the healthy sample showed the following mean (SD) times and errors: Task 1 = 24.701 (6.140) sec., 0 errors; Task 2 = 30.732 (9.744) sec., 0.126 (0.442) errors; Task 3 = 28.907 (11.221) sec., 0.175 (0.722) errors; Task 4 = 61.252 (20.781) sec., 1.918 (2.469) errors.

Taken together, both completion times and error rates confirm that Task 4 is substantially more difficult than the other tasks.

### Computing of the normative data

The optimal predictive models for each of the performance indexes considered are reported in Table 2. For detailed findings, including methodology and results, refer to Supplemental Materials (SM1).

**Table 2:** The optimal linear model for each performance index, with the Bayesian Information Criterion (BIC) and the R^2^ goodness of fit index. * = one observation is missing

Index	Model	BIC	*R*^2^
T4	y ~ 1 + [ln(education) + age^3]	3663.939	0.57
E4	y ~ 1 + [ln(education) + age^3]	2001.459	0.24
T3	y ~ 1 + [ln(education) + age^3]	3229.672	0.44
E3	y ~ 1 + age^3	969.171	0.08
T2	y ~ 1 + [ln(education) + age^3]	3064.843	0.48
E2*	y ~ 1 + age^2	547.885	0.03
T1	y ~ 1 + [ln(education) + age^3]	2728.287	0.38
T4 - T3	y ~ 1 + [√education + age]	3633.993	0.30
E4 - E3	y ~ 1 + [1/education + age^3]	1961.812	0.19
T4 – T2	y ~ 1 + [ln(education) + age]	3574.886	0.35
E4 – E2*	y ~ 1 + [ln(education) + ln(100 – age)]	1996.209	0.22
T3 – T1	y ~ 1 + [1/education + age^3]	2884.563	0.28

In all cases, the indices were impacted by education and age, with age transformed in almost all cases as the cubic elevation of age.

The correction scores are reported in Table 3. Tables detailing the means and standard deviations of raw scores for each SCWT test component are provided as SM2.

**Table 3:** Correction scores for the performance indexes of the Stroop test

STROOP T4:														
Age Edu	20–24	25–29	30–34	35–39	40–44	45–49	50–54	55–59	60–64	65–69	70–74	75–79	80–84	85–90
0–4	−6.82	−7.51	−8.50	−9.86	−11.63	−13.89	−16.67	−20.05	−24.08	−28.81	−34.31	−40.62	−47.81	−56.80
5–8	6.60	5.92	4.92	3.57	1.79	−0.46	−3.25	−6.63	−10.66	−15.39	−20.89	−27.20	−34.39	−43.37
9–13	12.59	11.91	10.92	9.56	7.78	5.53	2.74	−0.64	−4.67	−9.40	−14.89	−21.21	−28.39	−37.38
14–19	17.21	16.52	15.53	14.18	12.40	10.15	7.36	3.98	−0.05	−4.78	−10.28	−16.59	−23.78	−32.76
STROOP E4:														
Age Edu	20–24	25–29	30–34	35–39	40–44	45–49	50–54	55–59	60–64	65–69	70–74	75–79	80–84	85–90
0–4	−0.80	−0.85	−0.92	−1.02	−1.15	−1.31	−1.51	−1.76	−2.05	−2.40	−2.79	−3.25	−3.77	−4.43
5–8	0.39	0.34	0.27	0.17	0.04	−0.12	−0.33	−0.57	−0.86	−1.21	−1.60	−2.06	−2.58	−3.24
9–13	0.92	0.87	0.80	0.70	0.57	0.41	0.21	−0.04	−0.33	−0.67	−1.07	−1.53	−2.05	−2.71
14–19	1.33	1.28	1.21	1.11	0.98	0.82	0.62	0.37	0.08	−0.27	−0.66	−1.12	−1.64	−2.30
STROOP T3:														
Age Edu	20–24	25–29	30–34	35–39	40–44	45–49	50–54	55–59	60–64	65–69	70–74	75–79	80–84	85–90
0–4	−4.25	−4.56	−5.02	−5.64	−6.46	−7.49	−8.77	−10.32	−12.17	−14.35	−16.87	−19.77	−23.07	−27.19
5–8	2.70	2.39	1.93	1.31	0.50	−0.54	−1.82	−3.37	−5.22	−7.39	−9.92	−12.81	−16.11	−20.24
9–13	5.81	5.49	5.04	4.41	3.60	2.56	1.28	−0.27	−2.12	−4.29	−6.81	−9.71	−13.01	−17.14
14–19	8.20	7.88	7.43	6.81	5.99	4.96	3.68	2.12	0.28	−1.90	−4.42	−7.32	−10.62	−14.74
STROOP E3:														
Age	20–24	25–29	30–34	35–39	40–44	45–49	50–54	55–59	60–64	65–69	70–74	75–79	80–84	85–90
	0.19	0.18	0.17	0.14	0.12	0.08	0.04	−0.02	−0.08	−0.16	−0.24	−0.34	−0.46	−0.60
STROOP T2:														
Age Edu	20–24	25–29	30–34	35–39	40–44	45–49	50–54	55–59	60–64	65–69	70–74	75–79	80–84	85–90
0–4	−2.13	−2.43	−2.87	−3.46	−4.25	−5.24	−6.47	−7.96	−9.73	−11.82	−14.24	−17.02	−20.19	−24.15
5–8	3.17	2.86	2.43	1.83	1.05	0.05	−1.18	−2.66	−4.44	−6.52	−8.95	−11.73	−14.89	−18.85
9–13	5.53	5.23	4.79	4.19	3.41	2.42	1.19	−0.30	−2.08	−4.16	−6.58	−9.36	−12.53	−16.49
14–19	7.35	7.05	6.61	6.01	5.23	4.24	3.01	1.52	−0.26	−2.34	−4.76	−7.54	−10.71	−14.67
STROOP E2:														
Age	20–24	25–29	30–34	35–39	40–44	45–49	50–54	55–59	60–64	65–69	70–74	75–79	80–84	85–90
	6.14	5.79	5.29	4.61	3.71	2.56	1.15	−0.56	−2.60	−5.00	−7.78	−10.98	−14.62	−19.17
STROOP T1:														
Age Edu	20–24	25–29	30–34	35–39	40–44	45–49	50–54	55–59	60–64	65–69	70–74	75–79	80–84	85–90
0–4	−2.85	−3.00	−3.22	−3.53	−3.93	−4.44	−5.07	−5.83	−6.74	−7.81	−9.05	−10.47	−12.09	−14.12
5–8	1.11	0.95	0.73	0.42	0.02	−0.49	−1.12	−1.88	−2.79	−3.85	−5.09	−6.52	−8.14	−10.17
9–13	2.87	2.72	2.49	2.19	1.79	1.28	0.65	−0.11	−1.02	−2.09	−3.33	−4.75	−6.38	−8.40
14–19	4.23	4.08	3.85	3.55	3.15	2.64	2.01	1.25	0.34	−0.73	−1.97	−3.39	−5.02	−7.04
STROOP T4 - T3														
Age Edu	20–24	25–29	30–34	35–39	40–44	45–49	50–54	55–59	60–64	65–69	70–74	75–79	80–84	85–90
0–4	8.38	6.22	4.05	1.89	−0.28	−2.44	−4.61	−6.77	−8.94	−11.10	−13.27	−15.43	−17.60	−19.98
5–8	12.16	10.00	7.83	5.67	3.50	1.34	−0.83	−2.99	−5.16	−7.32	−9.49	−11.65	−13.82	−16.20
9–13	14.72	12.55	10.39	8.22	6.06	3.89	1.73	−0.44	−2.60	−4.77	−6.93	−9.10	−11.26	−13.65
14–19	17.20	15.03	12.87	10.70	8.54	6.37	4.21	2.04	−0.12	−2.29	−4.45	−6.62	−8.78	−11.16
STROOP E4 – E3														
Age Edu	20–24	25–29	30–34	35–39	40–44	45–49	50–54	55–59	60–64	65–69	70–74	75–79	80–84	85–90
0–4	−2.25	−2.29	−2.35	−2.42	−2.52	−2.65	−2.81	−3.00	−3.23	−3.50	−3.81	−4.17	−4.58	−5.09
5–8	0.26	0.22	0.16	0.09	−0.01	−0.14	−0.30	−0.49	−0.72	−0.99	−1.30	−1.66	−2.07	−2.58
9–13	0.72	0.68	0.62	0.54	0.44	0.31	0.16	−0.04	−0.26	−0.53	−0.84	−1.20	−1.61	−2.12
14–19	0.94	0.90	0.84	0.76	0.66	0.53	0.38	0.18	−0.04	−0.31	−0.62	−0.98	−1.39	−1.90
STROOP T4 – T2														
Age Edu	20–24	25–29	30–34	35–39	40–44	45–49	50–54	55–59	60–64	65–69	70–74	75–79	80–84	85–90
0–4	4.26	2.05	−0.16	−2.36	−4.57	−6.78	−8.99	−11.20	−13.41	−15.61	−17.82	−20.03	−22.24	−24.67
5–8	11.71	9.51	7.30	5.09	2.88	0.67	−1.54	−3.74	−5.95	−8.16	−10.37	−12.58	−14.79	−17.22
9–13	15.04	12.83	10.62	8.42	6.21	4.00	1.79	−0.42	−2.63	−4.83	−7.04	−9.25	−11.46	−13.89
14–19	17.60	15.40	13.19	10.98	8.77	6.56	4.35	2.15	−0.06	−2.27	−4.48	−6.69	−8.90	−11.33
STROOP E4 – E2														
Age Edu	20–24	25–29	30–34	35–39	40–44	45–49	50–54	55–59	60–64	65–69	70–74	75–79	80–84	85–90
0–4	−0.55	−0.68	−0.82	−0.97	−1.14	−1.32	−1.51	−1.73	−1.98	−2.26	−2.59	−2.98	−3.47	−4.19
5–8	0.63	0.50	0.36	0.20	0.04	−0.14	−0.34	−0.56	−0.80	−1.08	−1.41	−1.80	−2.29	−3.01
9–13	1.15	1.02	0.88	0.73	0.56	0.39	0.19	−0.03	−0.28	−0.56	−0.88	−1.28	−1.76	−2.49
14–19	1.56	1.43	1.29	1.13	0.97	0.79	0.59	0.37	0.13	−0.15	−0.48	−0.87	−1.36	−2.08
STROOP T3 - T1														
Age Edu	20–24	25–29	30–34	35–39	40–44	45–49	50–54	55–59	60–64	65–69	70–74	75–79	80–84	85–90
0–4	−4.81	−4.97	−5.19	−5.50	−5.90	−6.40	−7.03	−7.80	−8.70	−9.77	−11.01	−12.44	−14.06	−16.08
5–8	1.64	1.48	1.26	0.95	0.55	0.04	−0.59	−1.35	−2.26	−3.33	−4.56	−5.99	−7.61	−9.64
9–13	2.81	2.65	2.43	2.12	1.72	1.21	0.59	−0.18	−1.09	−2.15	−3.39	−4.82	−6.44	−8.47
14–19	3.37	3.22	2.99	2.69	2.29	1.78	1.15	0.39	−0.52	−1.59	−2.83	−4.25	−5.87	−7.90

The resulting equivalent scores are reported in Table 4, while the exact formulas to compute the corrected scores is reported in Table 5. However, an excel file for the automatic computation of correct scores is reported on Zenodo (see the Open Materials section).

**Table 4:** Internal Tolerance Limit, and Equivalent Scores cut-offs

	ITL	ES = 0	ES = 1	ES = 2	ES = 3	ES = 4
T4	78.36	> 86.20	> 67.64	> 59.92	> 55.13	≤55.13
E4	5.39	> 6.73	> 3.28	> 1.73	> 1.07	≤ 1.07
T3	39.20	> 48.03	> 31.74	> 28.54	> 25.76	≤ 25.76
E3	0.78	> 1.78	> 0.17	> 0.10	> 0.03	≤ 0.03
T2	37.41	> 44.98	> 33.30	> 30.32	> 27.94	≤ 27.94
E2	5.79	> 6.08	> 4.27	> 2.30	> 0.16	≤ 0.16
T1	30.29	> 32.85	> 32.85	> 27.18	> 25.05	≤ 25.05
T4 - T3	54.01	> 57.05	> 42.02	> 35.34	> 30.21	≤ 30.21
E4 - E3	5.26	> 6.46	> 3.22	> 1.57	> 0.82	≤ 0.82
T4 – T2	50.09	> 54.50	> 38.96	> 33.26	> 28.36	≤ 28.36
E4 – E2	5.42	> 6.70	> 3.18	> 1.70	> 1.07	≤ 1.07
T3 – T1	11.11	> 17.87	> 5.16	> 3.52	> 2.18	≤ 2.18

**Table 5:** the exact formula to compute the Corrected Score for each score of the test

Score	Exact formula
T4	Raw score - [−8.021699 * (√(education) - (3.16314629759675)) −30.9378348 * (ln(100-age) − 3.65101496467337)]
E4	Raw score - [−1.0398004 * (ln(education) - (2.3589319665131)) −2.2906369 * (ln(100-age) − 3.65101496467337)]
T3	Raw score - [−5.8994342 * (ln(education) - (2.3589319665131)) + 3.48e-05 * (age^3–170912.989789193)]
E3	Raw score - [1.2e-06 * (age^3–170912.989789193)]
T2	Raw score - [−4.4905181 * (ln(education) - (2.3589319665131)) + 3.34e-05 * (age^3–170912.989789193)]
E2	Raw score - [3.84e-05 * (age^3–170575.099525605)]
T1	Raw score - [−3.3535984 * (ln(education) - (2.3589319665131)) + 1.71e-05 * (age^3–170912.989789193)]
T4 - T3	Raw score - [−0.5593092 * (√(education) - (3.16314629759675)) + 7.1500263 * (age − 61.4862637)]
E4 - E3	Raw score - [7.1613468 * ((1/education) - (0.0999450851729517)) −1.703813 * (ln(100-age) − 3.65101496467337)]
T4 – T2	Raw score - [−6.3232321 * (ln(education) - (2.3589319665131)) + 0.4416765 * (age − 55.4955752)]
E4 – E2	Raw score - [−0.9990949 * (ln(education) - (2.35842458076661)) −1.9898895 * (ln(100-age) − 3.79641056233036)]
T3 – T1	Raw score - [18.6240033 * ((1/education) - (0.0945211210280571)) + 1.71e-05 * (age^3–170912.989789193)]

### Comparisons with Alzheimer’s disease patients’ score

To validate the new norms, we first analysed the test scores of patients diagnosed with Alzheimer’s Disease (AD) and Mild Cognitive Impairment (MCI) for T4, T3, E4, E3, T4 – T3 and E4 – E3 scores. We adjusted SCWT scores using the previously reported normative data to compute the corrected and equivalent scores (ES).

Next, we calculated the frequency and percentages of patients who received ES scores of 0, 1, 2, 3, and 4 based on the current norms. This frequency table can be found in Table 6.

**Table 6:** Distribution of Equivalent Scores (ES = 0–4) across participants with Alzheimer’s disease (AD) and mild cognitive impairment (MCI), reported as absolute frequencies and percentages. Panel A presents data from the combined AD + MCI sample; Panel B includes the AD group only; Panel C includes the MCI group only. Due to the retrospective nature of the dataset, Panels B and C report only indices derived from T4, T3, E4, and E3, as the remaining measures were not available. A) Total AD + MCI sample

A) Total AD + MCI sample						
	T4	E4	T3	E3	T4 - T3	E4 – E3
ES = 0	*N* = 126; % = 47.91	*N* = 132; % = 50.19	*N* = 57; % = 21.67	*N* = 32; % = 12.17	*N* = 120; % = 45.63	*N* = 123; % = 46.77
ES = 1	*N* = 68; % = 25.86	*N* = 42; % = 15.97	*N* = 70; % = 26.62	*N* = 44; % = 16.73	*N* = 57; % = 21.67	*N* = 49; % = 18.63
ES = 2	*N* = 19; % = 7.22	*N* = 34; % = 12.93	*N* = 31; % = 11.79	*N* = 0; % = 0	*N* = 22; % = 8.37	*N* = 34; % = 12.93
ES = 3	*N* = 15; % = 5.7	*N* = 10; % = 3.8	*N* = 19; % = 7.22	*N* = 2; % = 0.76	*N* = 14; % = 5.32	*N* = 14; % = 5.32
ES = 4	*N* = 35; % = 13.31	*N* = 45; % = 17.11	*N* = 86; % = 32.7	*N* = 185; % = 70.34	*N* = 50; % = 19.01	*N* = 43; % = 16.35
B) AD sample						
	T4	T3	E4	E3	T4 – T3	E4 – E3
ES = 0	*N* = 86; % = 69.35	*N* = 43; % = 34.68	*N* = 87; % = 70.16	*N* = 23; % = 18.55	*N* = 76; % = 61.29	*N* = 81; % = 65.32
ES = 1	*N* = 25; % = 20.16	*N* = 33; % = 26.61	*N* = 13; % = 10.48	*N* = 25; % = 20.16	*N* = 22; % = 17.74	*N* = 15; % = 12.1
ES = 2	*N* = 4; % = 3.23	*N* = 14; % = 11.29	*N* = 8; % = 6.45	*N* = 0; % = 0	*N* = 5; % = 4.03	*N* = 8; % = 6.45
ES = 3	*N* = 3; % = 2.42	*N* = 7; % = 5.65	*N* = 2; % = 1.61	*N* = 0; % = 0	*N* = 3; % = 2.42	*N* = 1; % = 0.81
ES = 4	*N* = 6; % = 4.84	*N* = 27; % = 21.77	*N* = 14; % = 11.29	*N* = 76; % = 61.29	*N* = 18; % = 14.52	*N* = 19; % = 15.32
C) MCI sample						
	T4	T3	E4	E3	T4 – T3	E4 – E3
ES = 0	*N* = 40; % = 28.78	*N* = 14; % = 10.07	*N* = 45; % = 32.37	*N* = 9; % = 6.47	*N* = 44; % = 31.65	*N* = 42; % = 30.22
ES = 1	*N* = 43; % = 30.94	*N* = 37; % = 26.62	*N* = 29; % = 20.86	*N* = 19; % = 13.67	*N* = 35; % = 25.18	*N* = 34; % = 24.46
ES = 2	*N* = 15; % = 10.79	*N* = 17; % = 12.23	*N* = 26; % = 18.71	*N* = 0; % = 0	*N* = 17; % = 12.23	*N* = 26; % = 18.71
ES = 3	*N* = 12; % = 8.63	*N* = 12; % = 8.63	*N* = 8; % = 5.76	*N* = 2; % = 1.44	*N* = 11; % = 7.91	*N* = 13; % = 9.35
ES = 4	*N* = 29; % = 20.86	*N* = 59; % = 42.45	*N* = 31; % = 22.3	*N* = 109; % = 78.42	*N* = 32; % = 23.02	*N* = 24; % = 17.27

Moreover, we computed Receiver Operating Characteristic (ROC) curves, the results of which are reported in Table 7. In the comparison between patients with Alzheimer’s disease (AD) and healthy participants, T4, E4, T4–T3, and E4–E3 showed AUC values greater than 0.80, indicating good discriminative ability, while T3 yielded an AUC of 0.742, reflecting acceptable discrimination. In the comparison between patients with mild cognitive impairment (MCI) and healthy participants, T4, E4, T4–T3, and E4–E3 demonstrated AUC values greater than 0.70, corresponding to acceptable discrimination. In contrast, when comparing patients with AD and MCI, only T4 and E4 achieved AUC values above 0.70. Overall, these findings highlight the greater difficulty in discriminating MCI from both healthy participants and patients with Alzheimer’s disease.

**Table 7:** Results of the Receiver Operating Characteristic (ROC) analyses. Reported values include the optimal cut-point determined by maximizing the Youden index. In the table are also reported accuracy, sensitivity, specificity, area under the curve (AUC), positive predictive value (PPV), and negative predictive value (NPV) for the T4, T3, E4, E3, T4–T3, and E4–E3 scores. **(A)** ROC analyses comparing patients with Alzheimer’s disease (AD) and healthy participants; scores above the cut-point indicate a higher likelihood of AD. **(B)** ROC analyses comparing patients with mild cognitive impairment (MCI) and healthy participants; scores above the cut-point indicate a higher likelihood of MCI. **(C)** ROC analyses comparing patients with AD and MCI; scores above the cut-point indicate a higher likelihood of AD. *** ^ AUC > 0.9; ** = AUC > 0.8; * = AUC > 0.7

A)	**AD/Healthy**	**Cut-Point**	**Youden**	**accuracy**	**sensitivity**	**specificity**	**AUC**	**PPV**	**NPV**
	T4 ***	73.702	0.746	0.884	0.855	0.892	0.929	0.684	0.957
	T3 *	31.639	0.434	0.767	0.629	0.805	0.742	0.470	0.888
	E4 **	4.338	0.689	0.875	0.790	0.898	0.870	0.681	0.940
	E3	0.000	0.170	0.569	0.613	0.558	0.492	0.275	0.840
	T4 - T3 **	44.263	0.633	0.840	0.774	0.858	0.837	0.600	0.933
	E4 - E3 **	4.744	0.668	0.882	0.750	0.918	0.834	0.715	0.930
B)	**MCI/Healthy**	**Cut-Point**	**Youden**	**accuracy**	**sensitivity**	**specificity**	**AUC**	**PPV**	**NPV**
	T4 *	65.540	0.437	0.756	0.647	0.790	0.753	0.486	0.879
	T3	31.276	0.202	0.702	0.410	0.792	0.596	0.377	0.814
	E4 *	2.206	0.404	0.719	0.669	0.735	0.730	0.437	0.878
	E3	0.000	0.327	0.607	0.770	0.558	0.623	0.349	0.887
	T4 - T3 *	43.617	0.399	0.777	0.554	0.845	0.706	0.524	0.860
	E4 - E3 *	1.976	0.418	0.723	0.683	0.735	0.744	0.442	0.883
C)	**AD/MCI**	**Cut-Point**	**Youden**	**accuracy**	**sensitivity**	**specificity**	**AUC**	**ppv**	**npv**
	T4 *	80.565	0.430	0.711	0.782	0.647	0.759	0.664	0.769
	T3	45.731	0.265	0.646	0.387	0.878	0.657	0.738	0.616
	E4 *	6.472	0.386	0.692	0.710	0.676	0.721	0.662	0.723
	E3	-Inf	0.000	0.529	0.000	1.000	0.408	-	0.529
	T4 - T3	61.328	0.314	0.662	0.573	0.741	0.683	0.664	0.660
	E4 - E3	4.744	0.362	0.677	0.750	0.612	0.688	0.633	0.733

Supplementary Table SM3 reports the percentiles computed from healthy participants, together with the corresponding percentages of patients with AD or MCI who exhibit worse scores.

## Discussion

The Stroop Colour and Word Test (SCWT) is a valid instrument used both in clinical and experimental settings to assess selective attention, information processing speed, cognitive flexibility, and inhibitory mechanisms, which are components of executive functions [37].

The present article had three main objectives: (i) to introduce a new version of the Stroop Colour and Word Test; (ii) to provide normative data from a large sample of healthy Italian adults, stratified by age, sex, and education; and (iii) to offer preliminary sensitivity data based on a small sample of patients with Alzheimer’s disease.

### The impact of demographic variables on SCWT performance

To compute normative data for the SCWT, we examined the effects of age, sex, and education on test performance. The analyses showed that performance was consistently influenced by both education and age, with the exception of indices E3 and E2 (which were affected only by age) and E1 (which was always equal to 0 and thus unaffected by either variable).

A notable finding was that the effect of education followed a non-linear pattern. In seven out of twelve indices, the relationship was best captured by a natural logarithmic transformation; in the remaining cases, other adjustments such as square root (one index) or reciprocal (two indices) transformations were required. This suggests that education does not impact SCWT performance in a simple linear way.

Age effects, by contrast, were observed in all indices except E1. In most cases (8 out of 12), a cubic transformation best accounted for the age effect; in one case a quadratic transformation was applied, in another the natural logarithm of 100 – age, and in two cases no transformation was necessary. This indicates that age-related changes in performance also follow complex, predominantly non-linear trajectories.

As expected, increasing age is linked to worse scores, while increasing education is linked to better performances. Similarly, several previous studies have shown that age and education influence performance, whereas sex appears to be non-significant in some cases [25, 26, 38] but in others [7, 28] females performed significantly better than males.

### The heterogeneity of SCWT in the Italian context

Previous published Italian adaptation of the SCWT show a marked heterogeneity that can be observed across the various standardization studies conducted over time.

An analysis of the Italian SCWT versions [7, 26–28, 38, 39] reveals significant variability in both the number of stimuli (from 30 to 100) and the number of colours used (from 3 to 5). Methodologically, studies differ in task types, scoring procedures—some count errors or correct responses on a fixed set of stimuli, while others do so within a time limit regardless of total stimuli presented [9, 40]—and in the calculation of composite indices using varying formulas [26, 38], often combining colour-naming and word-reading tasks.

Specifically, with regard to the type of response and conditions assessed (see [37], for a review), some studies [26, 39] investigated two types of interference effects: response speed (time variable) and naming accuracy (error variable). This distinction has been criticized by some authors as ambiguous. Indeed, in some studies, only one of the two interference measures (namely, the number of errors) was able to discriminate between patients with frontal deficits and healthy controls [41].

Barbarotto et al. [28] and subsequently Brugnolo et al. [7] attempted to address this issue by combining the two interference measures, asking participants to name as many stimuli as possible within a fixed time (30 s), recording the number of errors.

Brugnolo [7] used a 100-item task but recorded the number of correct responses obtained in the first 30 s for each table, generating three scores: word items (WI), colour items (CI), and colour-word items (CWI). The total time taken to complete each table was also recorded, producing three additional scores: word time (WT), colour time (CT), and colour-word time (CWT).

Nevertheless, these indices do not allow for identifying the underlying cause of altered performance; for example, a subject with ideational slowing cannot be distinguished from one with impaired inhibition of automatic responses, as errors are not directly analysed or counted. Moreover, Brugnolo does not assess the time required to name the ink colour in the incongruent condition—typically the most informative clinical variable, as it tends to produce the highest error rate.

For these reasons, we proposed a new 50-item version of the test, presented on an A4 sheet for ease of use. This item count aligns with clinical recommendations [19, 25] and allows reliable assessment of processing speed and response inhibition in older adults [25]. Errors and completion times were recorded separately, and specific indices were calculated to detect all potential interferences within tasks targeting the same cognitive function.

Although the classical Stroop effect is related to the difference between task types (automatic word reading vs. less automated colour naming), the presence of congruent (black-and-white words and coloured dots) and incongruent stimuli (words written in ink colours incongruent with their meaning) introduces variability in response that must be recorded and analysed.

Therefore, Task 1 (reading words in black ink) was compared, in terms of both errors and reading time, with Task 3 (reading words in incongruent ink colours), allowing measurement of colour interference on a reading task. Conversely, comparing Task 2 (naming coloured dots) and Task 4 (naming the ink colour of incongruent colour-words) allows assessment of word interference in a colour-naming task.

Finally, the classical comparison between Task 3 (incongruent colour-word reading) and Task 4 (colour naming of incongruent colour-words) allows the analysis of the cognitive cost associated with the nature of the required task, that is, comparing an automatic task (reading) with a controlled task (naming) [37, 42].

These comparisons allow for verification of all possible interactions between stimulus type and task type.

From the results, it emerged that the first three tasks (word reading, colour naming and incongruent colour-word reading, respectively) showed that the tasks were simple, indeed the error rate was extremely low (E1 was consistently equal to zero) and with short times. Times and errors increase in Task 4 (colour naming of incongruent colour-words), confirming this as the most cognitively demanding.

### ROC analysis of the SCWT

A known limitation of neuropsychological testing is that normative cut-offs are typically based on healthy participants, ensuring specificity but leaving sensitivity uncertain, as pathological performance is not represented. Valid sensitivity estimates require large, representative clinical samples. Additionally, since various neurological and psychiatric conditions affect executive functions differently, pathology-specific cut-offs are likely needed instead of a single universal threshold.

To this end, we also retrieved retrospective data from patients with AD and MCI, allowing us to perform ROC analyses and to identify optimal cut-off values for the T4, T3, E4, E3, T4–T3, and E4–E3 scores by maximizing the Youden index. ROC analyses were conducted by comparing patients with AD to healthy participants, patients with MCI to healthy participants, and patients with AD to those with MCI. Consistent with previous literature, the T4 and E4 indices demonstrated the best discriminative performance, achieving AUC values ≥ 0.70 across all three comparisons.

However, in the comparison between AD and MCI, specificity values for both indices were below 0.70, whereas in the comparison between MCI and healthy participants, sensitivity values did not exceed 0.70. Given these limitations, and to support alternative threshold selection, Supplementary Table SM3 reports the percentiles of the T4 and E4 scores together with the corresponding proportions of patients with AD and MCI who obtained worse scores.

In summary, AUC values suggest the difficulty in discriminating between patients and healthy participants, and more strikingly, between AD and MCI. This should warn the neuropsychologist to never rely solely on questionnaire cut-off scores; rather, each evaluation should be grounded in a comprehensive, multidisciplinary assessment that integrates psychometric results with clinical observations, qualitative information, and convergent reports from caregivers or other individuals closely involved in the patient’s daily functioning.

Future research should expand on this work by including larger, more diverse clinical groups, covering Alzheimer’s disease (AD) and other conditions with executive impairment like frontotemporal dementia, Parkinson’s disease, and traumatic brain injury. This would enable development of pathology-specific cut-offs, improve sensitivity estimates, and enhance the SCWT’s clinical utility. Longitudinal studies could also determine whether SCWT performance tracks disease progression, supporting its role in monitoring cognitive decline.

This standardization includes all four tasks, recording both time and errors separately, allowing clinicians to detect pathological patterns related to interference or automaticity. The calculated indices help identify specific performance profiles—for instance, ideational slowing may affect all tasks, while prolonged time in one task may indicate task-specific processing issues.

Key strengths of this study include a larger sample size than prior Italian standardizations, a wide age range (20–90 years), and separate analyses of speed and accuracy across tasks. We disagree with using a global interference index and instead advocate for separate evaluation of accuracy and speed, as they reflect distinct executive processes and often show dissociation in clinical settings [40].

Preliminary findings in patients with mild to moderate AD suggest high sensitivity and specificity, particularly for completion time. The main limitation is the verbal nature of the material, restricting use to Italian speakers, and the regional focus on the Marche population, which may limit generalizability.

In conclusion, this study offers updated normative data for a 50-item Stroop Test version in Italian adults aged 20–90, suitable for both clinical and research use, and effective in identifying selective attention deficits in AD patients.

### Open materials

An Excel file for the automatic correction of raw scores is provided as an Excel file as Supplementary Materials 3 and at the following link: 10.5281/zenodo.18057327.

## Supplementary Information

Below is the link to the electronic supplementary material.

Supplementary Material 1 (DOCX 112 KB)

## Data Availability

Data are available upon reasonable request.

## Declarations

### Provenance and peer review

Not commissioned; externally peer reviewed.
