# Supplementary material for: An updated italian normative data for a short version of the stroop colour word test
Source: Neurol Sci. 2026 Mar 25;47(4):370. doi: 10.1007/s10072-026-08918-4 (PMC13013156; doi:10.1007/s10072-026-08918-4)
Supplement: Supplementary file 1 — Supplementary Material 1 (DOCX 112 KB) [file 10072_2026_8918_MOESM1_ESM.docx]

# Supplementary Materials

## SM1 The impact of Age, Education and Sex on the indexes and computation of correction scores.

Data from the healthy sample were analysed by means of Linear models using different combinations of Sex, Age and Education. In particular, models could be characterised from no predictors to more complex models by adding Sex and for Age and Education. For the two latter, linear, square root, quadratic, cubic, natural logarithm, inverse transformation, and for age the natural logarithm of 100 - age were used, for a total of 111 models.

The fitted models where then compared by means of Bayesian Information Criterion (BIC) in order to select the model that best predicts the data. The best model for a specific index was identified as the model in which the BIC is less than 2 points compared to all the other models for that index or, if there are more models with a low BIC less than 2 points, the simplest model. In Table SM1 there are all the BICs of all the computed models.

|  | T4 | E4 | T3 | E3 | T2 | E2 | T1 | T4 – T3 | E4 – E3 | T4 – T1 | T4 – T2 | E4 – E2 | T3 – T1 |  |
| --- | --- | --- | --- | --- | --- | --- | --- | --- | --- | --- | --- | --- | --- | --- |
| No Effect | 4036.734 | 2111.108 | 3479.66 | 999.666 | 3352.063 | 554.066 | 2934.553 | 3781.068 | 2046.428 | 3881.17 | 3760.68 | 2094.794 | 3018.947 |  |
| ~ 1 + sex | 4042.57 | 2115.046 | 3484.738 | 1005.714 | 3358.137 | 559.492 | 2939.349 | 3787.181 | 2050.476 | 3887.234 | 3766.447 | 2099.04 | 3024.624 |  |
| ~ 1 + age | 3737.461 | 2028.301 | 3313.058 | 981.623 | 3151.893 | 549.341 | 2801.347 | 3640.686 | 1981.601 | 3637.39 | 3592.299 | 2021.45 | 2923.077 |  |
| ~ 1 + age + sex | 3743.517 | 2030.933 | 3318.386 | 987.716 | 3157.988 | 554.596 | 2806.391 | 3646.677 | 1984.599 | 3643.483 | 3598.279 | 2024.537 | 2928.956 |  |
| ~ 1 + √(age) | 3756.373 | 2034.206 | 3331.172 | 985.321 | 3174.016 | 550.402 | 2815.458 | 3642.432 | 1985.162 | 3647.942 | 3595.441 | 2026.458 | 2934.07 |  |
| ~ 1 + √(age) + sex | 3762.444 | 2036.835 | 3336.554 | 991.414 | 3180.108 | 555.656 | 2820.558 | 3648.406 | 1988.139 | 3654.027 | 3601.441 | 2029.54 | 2939.962 |  |
| ~ 1 + age^2 | 3717.834 | 2019.798 | 3284.234 | 974.752 | 3116.579 | **547.885** | 2778.917 | 3644.41 | 1977.222 | 3630.768 | 3594.958 | 2014.245 | 2905.674 |  |
| ~ 1 + age^2 + sex | 3723.857 | 2022.446 | 3289.463 | 980.843 | 3122.68 | 553.148 | 2783.859 | 3650.431 | 1980.268 | 3636.874 | 3600.903 | 2017.355 | 2911.526 |  |
| ~ 1 + age^3 | 3719.069 | 2015.284 | 3267.359 | **969.171** | 3095.562 | 547.327 | 2765.132 | 3653.867 | 1975.801 | 3638.742 | 3605.28 | 2010.359 | 2895.72 |  |
| ~ 1 + age^3 + sex | 3725.069 | 2017.946 | 3272.522 | 975.262 | 3101.666 | 552.597 | 2770.006 | 3659.906 | 1978.884 | 3644.853 | 3611.203 | 2013.489 | 2901.554 |  |
| ~ 1 + ln(age) | 3780.61 | 2041.092 | 3350.677 | 988.99 | 3197.813 | 551.628 | 2830.807 | 3647.14 | 1989.639 | 3663.356 | 3602.064 | 2032.326 | 2945.91 |  |
| ~ 1 + ln(age) + sex | 3786.694 | 2043.729 | 3356.107 | 995.082 | 3203.903 | 556.881 | 2835.959 | 3653.096 | 1992.602 | 3669.432 | 3608.083 | 2035.411 | 2951.813 |  |
| ~ 1 + 1/age | 3837.947 | 2056.575 | 3389.311 | 995.53 | 3244.875 | 554.228 | 2861.844 | 3664.832 | 2000.602 | 3704.519 | 3624.492 | 2045.658 | 2969.273 |  |
| ~ 1 + 1/age + sex | 3844.046 | 2059.268 | 3394.808 | 1001.62 | 3250.965 | 559.491 | 2867.071 | 3670.76 | 2003.573 | 3710.58 | 3630.54 | 2048.787 | 2975.188 |  |
| ~ 1 + ln(100-age) | 3717.615 | 2014.826 | 3276.626 | 971.347 | 3104.843 | 548.107 | 2769.734 | 3648.317 | 1974.631 | 3635.193 | 3600.199 | 2009.528 | 2903.391 |  |
| ~ 1 + ln(100-age) + sex | 3723.655 | 2017.36 | 3281.892 | 977.443 | 3110.935 | 553.364 | 2774.711 | 3654.329 | 1977.601 | 3641.294 | 3606.159 | 2012.545 | 2909.261 |  |
| ~ 1 + education | 3935.776 | 2071.513 | 3402.737 | 996.85 | 3277.658 | 559.175 | 2862.566 | 3739.675 | 2012.015 | 3805.998 | 3701.702 | 2056.681 | 2979.842 |  |
| ~ 1 + education + sex | 3941.889 | 2073.921 | 3408.48 | 1002.954 | 3283.692 | 564.509 | 2868.102 | 3745.622 | 2014.693 | 3812.046 | 3707.781 | 2059.579 | 2985.847 |  |
| ~ 1 + education + age | 3694.078 | 2017.451 | 3283.426 | 985.748 | 3126.613 | 555.428 | 2772.537 | 3634.087 | 1972 | 3613.941 | 3576.193 | 2010.541 | 2914.02 |  |
| ~ 1 + education + age + sex | 3700.183 | 2019.137 | 3289.142 | 991.856 | 3132.555 | 560.695 | 2778.031 | 3639.923 | 1974.149 | 3619.885 | 3582.286 | 2012.79 | 2920.041 |  |
| ~ 1 + education + √(age) | 3713.469 | 2022.858 | 3300.491 | 989.079 | 3147.757 | 556.503 | 2785.556 | 3635.995 | 1975.26 | 3624.584 | 3579.525 | 2015.088 | 2924.041 |  |
| ~ 1 + education + √(age) + sex | 3719.567 | 2024.538 | 3306.244 | 995.188 | 3153.69 | 561.763 | 2791.092 | 3641.809 | 1977.388 | 3630.508 | 3585.624 | 2017.33 | 2930.071 |  |
| ~ 1 + education + age^2 | 3670.779 | 2008.928 | 3254.383 | 979.303 | 3090.841 | 553.962 | 2750.251 | 3636.36 | 1967.379 | 3605.056 | 3576.949 | 2003.314 | 2897.236 |  |
| ~ 1 + education + age^2 + sex | 3676.891 | 2010.611 | 3260.035 | 985.409 | 3096.797 | 559.239 | 2755.674 | 3642.228 | 1969.556 | 3611.029 | 3583.031 | 2005.567 | 2903.24 |  |
| ~ 1 + education + age^3 | 3666.303 | 2003.455 | 3235.035 | 973.86 | 3067.025 | 553.417 | 2734.642 | 3643.463 | 1964.97 | 3609.085 | 3584.167 | 1998.538 | 2886.568 |  |
| ~ 1 + education + age^3 + sex | 3672.416 | 2005.11 | 3240.654 | 979.966 | 3072.98 | 558.698 | 2740.028 | 3649.344 | 1967.148 | 3615.068 | 3590.245 | 2000.775 | 2892.565 |  |
| ~ 1 + education + ln(age) | 3737.036 | 2028.842 | 3318.085 | 992.263 | 3169.671 | 557.738 | 2799.047 | 3640.375 | 1979.091 | 3639.214 | 3585.68 | 2020.119 | 2934.418 |  |
| ~ 1 + education + ln(age) + sex | 3743.126 | 2030.518 | 3323.874 | 998.372 | 3175.593 | 562.993 | 2804.624 | 3646.163 | 1981.198 | 3645.114 | 3591.785 | 2022.355 | 2940.458 |  |
| ~ 1 + education + 1/age | 3789.587 | 2041.339 | 3350.607 | 997.517 | 3210.508 | 560.308 | 2824.3 | 3655.797 | 1987.722 | 3676.132 | 3605.069 | 2030.673 | 2953.56 |  |
| ~ 1 + education + 1/age + sex | 3795.657 | 2043.022 | 3356.449 | 1003.626 | 3216.419 | 565.556 | 2829.94 | 3661.534 | 1989.799 | 3681.991 | 3611.181 | 2032.913 | 2959.613 |  |
| ~ 1 + education + ln(100-age) | 3667.508 | 2003.707 | 3245.433 | 976.01 | 3077.691 | 554.199 | 2740.262 | 3639.092 | 1964.48 | 3607.354 | 3580.54 | 1998.408 | 2894.343 |  |
| ~ 1 + education + ln(100-age) + sex | 3673.613 | 2005.257 | 3251.115 | 982.118 | 3083.613 | 559.466 | 2745.713 | 3644.94 | 1966.568 | 3613.303 | 3586.63 | 2000.554 | 2900.36 |  |
| ~ 1 + √(education) | 3928.145 | 2067.822 | 3395.106 | 997.132 | 3271.6 | 559.134 | 2854.689 | 3737.473 | 2008.396 | 3801.278 | 3697.576 | 2053.07 | 2976.103 |  |
| ~ 1 + √(education) + sex | 3934.247 | 2069.885 | 3400.966 | 1003.24 | 3277.563 | 564.45 | 2860.369 | 3743.354 | 2010.755 | 3807.262 | 3703.68 | 2055.659 | 2982.154 |  |
| ~ 1 + √(education) + age | 3691.154 | 2015.964 | 3279.741 | 986.062 | 3124.388 | 555.419 | 2768.376 | **3633.993** | 1970.365 | 3612.989 | 3575.13 | 2009.02 | 2912.639 |  |
| ~ 1 + √(education) + age + sex | 3697.238 | 2017.417 | 3285.543 | 992.171 | 3130.271 | 560.69 | 2773.979 | 3639.791 | 1972.295 | 3618.883 | 3581.235 | 2011.057 | 2918.686 |  |
| ~ 1 + √(education) + √(age) | 3710.17 | 2021.224 | 3296.489 | 989.39 | 3145.208 | 556.498 | 2781.1 | 3635.791 | 1973.511 | 3623.368 | 3578.298 | 2013.431 | 2922.467 |  |
| ~ 1 + √(education) + √(age) + sex | 3716.242 | 2022.666 | 3302.325 | 995.499 | 3151.078 | 561.762 | 2786.744 | 3641.564 | 1975.415 | 3629.236 | 3584.407 | 2015.455 | 2928.523 |  |
| ~ 1 + √(education) + age^2 | 3668.36 | 2007.634 | 3251.121 | 979.625 | 3089.076 | 553.947 | 2746.478 | 3636.401 | 1965.884 | 3604.449 | 3576.065 | 2001.975 | 2896.118 |  |
| ~ 1 + √(education) + age^2 + sex | 3674.457 | 2009.092 | 3256.864 | 985.732 | 3094.977 | 559.229 | 2752.015 | 3642.232 | 1967.846 | 3610.375 | 3582.163 | 2004.022 | 2902.149 |  |
| ~ 1 + √(education) + age^3 | 3664.091 | 2002.25 | 3231.952 | 974.197 | 3065.501 | 553.403 | 2731.028 | 3643.546 | 1963.523 | 3608.611 | 3583.309 | 1997.281 | 2885.572 |  |
| ~ 1 + √(education) + age^3 + sex | 3670.191 | 2003.679 | 3237.666 | 980.303 | 3071.401 | 558.69 | 2736.531 | 3649.388 | 1965.484 | 3614.548 | 3589.405 | 1999.312 | 2891.597 |  |
| ~ 1 + √(education) + ln(age) | 3733.289 | 2027.03 | 3313.701 | 992.568 | 3166.74 | 557.737 | 2794.233 | 3640.03 | 1977.198 | 3637.673 | 3584.238 | 2018.294 | 2932.612 |  |
| ~ 1 + √(education) + ln(age) + sex | 3739.346 | 2028.458 | 3319.571 | 998.678 | 3172.596 | 562.993 | 2799.916 | 3645.773 | 1979.073 | 3643.513 | 3590.35 | 2020.305 | 2938.677 |  |
| ~ 1 + √(education) + 1/age | 3784.812 | 2039.098 | 3345.35 | 997.805 | 3206.716 | 560.308 | 2818.661 | 3655.08 | 1985.466 | 3673.812 | 3603.062 | 2028.446 | 2951.222 |  |
| ~ 1 + √(education) + 1/age + sex | 3790.837 | 2040.511 | 3351.27 | 1003.916 | 3212.55 | 565.552 | 2824.407 | 3660.763 | 1987.294 | 3679.598 | 3609.175 | 2030.442 | 2957.299 |  |
| ~ 1 + √(education) + ln(100-age) | 3665.192 | 2002.484 | 3242.251 | 976.337 | 3076.049 | 554.187 | 2736.586 | 3639.161 | 1963.032 | 3606.812 | 3579.669 | 1997.139 | 2893.263 |  |
| ~ 1 + √(education) + ln(100-age) + sex | 3671.277 | 2003.808 | 3248.021 | 982.445 | 3081.911 | 559.459 | 2742.148 | 3644.968 | 1964.904 | 3612.709 | 3585.773 | 1999.078 | 2899.305 |  |
| ~ 1 + education^2 | 3952.798 | 2079.087 | 3417.897 | 997.113 | 3290.285 | 559.344 | 2878.095 | 3745.621 | 2019.016 | 3817.339 | 3711.562 | 2063.974 | 2987.293 |  |
| ~ 1 + education^2 + sex | 3958.87 | 2082.034 | 3423.412 | 1003.205 | 3296.387 | 564.711 | 2883.362 | 3751.656 | 2022.183 | 3823.445 | 3717.563 | 2067.35 | 2993.202 |  |
| ~ 1 + education^2 + age | 3701.599 | 2020.498 | 3290.667 | 985.488 | 3131.435 | 555.431 | 2780.588 | 3635.168 | 1975.035 | 3617.288 | 3579.415 | 2013.565 | 2916.716 |  |
| ~ 1 + education^2 + age + sex | 3707.711 | 2022.545 | 3296.23 | 991.593 | 3137.455 | 560.694 | 2785.895 | 3641.067 | 1977.516 | 3623.305 | 3585.474 | 2016.138 | 2922.686 |  |
| ~ 1 + education^2 + √(age) | 3721.404 | 2026.118 | 3308.184 | 988.861 | 3153.046 | 556.503 | 2794.035 | 3637.21 | 1978.455 | 3628.261 | 3582.936 | 2018.313 | 2927.038 |  |
| ~ 1 + education^2 + √(age) + sex | 3727.518 | 2028.167 | 3313.789 | 994.966 | 3159.06 | 561.761 | 2799.388 | 3643.089 | 1980.92 | 3634.263 | 3589.006 | 2020.885 | 2933.019 |  |
| ~ 1 + education^2 + age^2 | 3677.976 | 2011.742 | 3261.155 | 978.985 | 3095.133 | 553.97 | 2757.856 | 3637.366 | 1970.264 | 3608.124 | 3580.089 | 2006.119 | 2899.574 |  |
| ~ 1 + education^2 + age^2 + sex | 3684.082 | 2013.782 | 3266.644 | 985.088 | 3101.161 | 559.241 | 2763.085 | 3643.294 | 1972.77 | 3614.163 | 3586.131 | 2008.693 | 2905.526 |  |
| ~ 1 + education^2 + age^3 | 3673.7 | 2006.234 | 3241.809 | 973.501 | 3071.225 | 553.424 | 2742.238 | 3644.579 | 1967.869 | 3612.268 | 3587.508 | 2001.308 | 2888.812 |  |
| ~ 1 + education^2 + age^3 + sex | 3679.802 | 2008.253 | 3247.255 | 979.604 | 3077.255 | 558.7 | 2747.42 | 3650.524 | 1970.383 | 3618.318 | 3593.542 | 2003.871 | 2894.754 |  |
| ~ 1 + education^2 + ln(age) | 3745.561 | 2032.386 | 3326.376 | 992.097 | 3175.561 | 557.737 | 2808.093 | 3641.798 | 1982.509 | 3643.357 | 3589.395 | 2023.609 | 2937.798 |  |
| ~ 1 + education^2 + ln(age) + sex | 3751.674 | 2034.441 | 3332.019 | 998.203 | 3181.57 | 562.993 | 2813.489 | 3647.656 | 1984.96 | 3649.343 | 3595.476 | 2026.184 | 2943.789 |  |
| ~ 1 + education^2 + 1/age | 3799.705 | 2045.621 | 3360.402 | 997.481 | 3217.892 | 560.321 | 2834.782 | 3657.858 | 1991.743 | 3681.551 | 3609.709 | 2034.852 | 2957.882 |  |
| ~ 1 + education^2 + 1/age + sex | 3805.814 | 2047.712 | 3366.1 | 1003.585 | 3223.899 | 565.577 | 2840.241 | 3663.678 | 1994.189 | 3687.51 | 3615.805 | 2037.456 | 2963.884 |  |
| ~ 1 + education^2 + ln(100-age) | 3674.877 | 2006.47 | 3252.255 | 975.672 | 3081.97 | 554.205 | 2747.864 | 3640.162 | 1967.344 | 3610.531 | 3583.812 | 2001.157 | 2896.694 |  |
| ~ 1 + education^2 + ln(100-age) + sex | 3680.988 | 2008.381 | 3257.778 | 981.778 | 3087.975 | 559.467 | 2753.123 | 3646.075 | 1969.764 | 3616.554 | 3589.866 | 2003.626 | 2902.661 |  |
| ~ 1 + education^3 | 3967.894 | 2085.509 | 3430.536 | 997.947 | 3301.211 | 559.535 | 2890.733 | 3751.457 | 2024.699 | 3827.923 | 3720.648 | 2070.095 | 2993.689 |  |
| ~ 1 + education^3 + sex | 3973.897 | 2088.796 | 3435.878 | 1004.027 | 3307.325 | 564.925 | 2895.807 | 3757.533 | 2028.17 | 3834.036 | 3726.573 | 2073.768 | 2999.518 |  |
| ~ 1 + education^3 + age | 3708.812 | 2023.061 | 3296.671 | 985.58 | 3135.769 | 555.424 | 2786.979 | 3636.589 | 1977.404 | 3620.985 | 3582.696 | 2016.059 | 2919.043 |  |
| ~ 1 + education^3 + age + sex | 3714.909 | 2025.333 | 3302.128 | 991.68 | 3141.827 | 560.685 | 2792.163 | 3642.527 | 1980.087 | 3627.039 | 3588.726 | 2018.83 | 2924.976 |  |
| ~ 1 + education^3 + √(age) | 3728.733 | 2028.805 | 3314.439 | 989.006 | 3157.642 | 556.495 | 2800.672 | 3638.681 | 1980.911 | 3632.091 | 3586.279 | 2020.923 | 2929.559 |  |
| ~ 1 + education^3 + √(age) + sex | 3734.838 | 2031.082 | 3319.941 | 995.106 | 3163.695 | 561.753 | 2805.904 | 3644.6 | 1983.579 | 3638.134 | 3592.323 | 2023.696 | 2935.503 |  |
| ~ 1 + education^3 + age^2 | 3685.375 | 2014.215 | 3267.024 | 979.008 | 3099.292 | 553.965 | 2764.097 | 3638.855 | 1972.59 | 3611.872 | 3583.49 | 2008.526 | 2901.715 |  |
| ~ 1 + education^3 + age^2 + sex | 3691.457 | 2016.482 | 3272.399 | 985.107 | 3105.357 | 559.233 | 2769.195 | 3644.822 | 1975.3 | 3617.945 | 3589.496 | 2011.3 | 2907.628 |  |
| ~ 1 + education^3 + age^3 | 3681.663 | 2008.772 | 3247.917 | 973.491 | 3075.574 | 553.42 | 2748.668 | 3646.288 | 1970.277 | 3616.368 | 3591.241 | 2003.771 | 2890.98 |  |
| ~ 1 + education^3 + age^3 + sex | 3687.736 | 2011.025 | 3253.241 | 979.59 | 3081.642 | 558.695 | 2753.713 | 3652.271 | 1973.001 | 3622.45 | 3597.235 | 2006.54 | 2896.881 |  |
| ~ 1 + education^3 + ln(age) | 3753.167 | 2035.257 | 3333.011 | 992.308 | 3180.541 | 557.732 | 2815.096 | 3643.385 | 1985.103 | 3647.443 | 3592.898 | 2026.391 | 2940.586 |  |
| ~ 1 + education^3 + ln(age) + sex | 3759.277 | 2037.544 | 3338.554 | 998.409 | 3186.592 | 562.988 | 2820.372 | 3649.286 | 1987.761 | 3653.472 | 3598.956 | 2029.17 | 2946.54 |  |
| ~ 1 + education^3 + 1/age | 3808.298 | 2049.017 | 3368.109 | 997.857 | 3223.943 | 560.334 | 2842.815 | 3659.885 | 1994.754 | 3686.481 | 3613.824 | 2038.123 | 2961.373 |  |
| ~ 1 + education^3 + 1/age + sex | 3814.411 | 2051.356 | 3373.708 | 1003.957 | 3229.994 | 565.595 | 2848.154 | 3665.754 | 1997.418 | 3692.491 | 3619.902 | 2040.944 | 2967.337 |  |
| ~ 1 + education^3 + ln(100-age) | 3682.662 | 2008.959 | 3258.303 | 975.679 | 3086.276 | 554.2 | 2754.217 | 3641.783 | 1969.698 | 3614.526 | 3587.424 | 2003.572 | 2898.906 |  |
| ~ 1 + education^3 + ln(100-age) + sex | 3688.755 | 2011.102 | 3263.713 | 981.781 | 3092.323 | 559.461 | 2759.348 | 3647.737 | 1972.326 | 3620.587 | 3593.444 | 2006.245 | 2904.835 |  |
| ~ 1 + ln(education) | 3922.868 | 2064.845 | 3388.622 | 997.783 | 3266.806 | 559.148 | 2848.056 | 3736.538 | 2005.233 | 3798.429 | 3695.078 | 2050.093 | 2972.842 |  |
| ~ 1 + ln(education) + sex | 3928.927 | 2066.541 | 3394.582 | 1003.893 | 3272.673 | 564.447 | 2853.867 | 3742.345 | 2007.247 | 3804.331 | 3701.191 | 2052.349 | 2978.926 |  |
| ~ 1 + ln(education) + age | 3689.712 | 2014.778 | 3276.564 | 986.487 | 3122.726 | 555.399 | 2764.833 | 3634.431 | 1968.883 | 3613.008 | **3574.886** | 2007.758 | 2911.379 |  |
| ~ 1 + ln(education) + age + sex | 3695.762 | 2015.986 | 3282.446 | 992.596 | 3128.539 | 560.678 | 2770.542 | 3640.193 | 1970.572 | 3618.846 | 3580.998 | 2009.566 | 2917.449 |  |
| ~ 1 + ln(education) + √(age) | 3708.239 | 2019.875 | 3292.949 | 989.832 | 3143.18 | 556.486 | 2777.226 | 3636.095 | 1971.896 | 3623.063 | 3577.848 | 2012.015 | 2920.998 |  |
| ~ 1 + ln(education) + √(age) + sex | 3714.27 | 2021.062 | 3298.863 | 995.942 | 3148.974 | 561.756 | 2782.973 | 3641.829 | 1973.552 | 3628.871 | 3583.962 | 2013.803 | 2927.076 |  |
| ~ 1 + ln(education) + age^2 | 3667.705 | 2006.693 | 3248.499 | 980.027 | 3088.008 | 553.917 | 2743.437 | 3637.054 | 1964.589 | 3604.971 | 3576.119 | 2000.941 | 2895.17 |  |
| ~ 1 + ln(education) + age^2 + sex | 3673.775 | 2007.916 | 3254.328 | 986.133 | 3093.844 | 559.21 | 2749.083 | 3642.85 | 1966.317 | 3610.848 | 3582.226 | 2002.769 | 2901.225 |  |
| ~ 1 + ln(education) + age^3 | **3663.939** | **2001.459** | **3229.672** | 974.598 | **3064.843** | 553.374 | **2728.287** | 3644.34 | 1962.326 | 3609.448 | 3583.522 | 1996.386 | 2884.809 |  |
| ~ 1 + ln(education) + age^3 + sex | 3670.014 | 2002.656 | 3235.474 | 980.704 | 3070.679 | 558.671 | 2733.902 | 3650.147 | 1964.055 | 3615.336 | 3589.629 | 1998.2 | 2890.859 |  |
| ~ 1 + ln(education) + ln(age) | 3730.824 | 2025.492 | 3309.755 | 993.03 | 3164.308 | 557.733 | 2789.985 | 3640.178 | 1975.423 | 3637.003 | 3583.543 | 2016.7 | 2930.903 |  |
| ~ 1 + ln(education) + ln(age) + sex | 3736.833 | 2026.653 | 3315.699 | 999.141 | 3170.081 | 562.993 | 2795.769 | 3645.879 | 1977.042 | 3642.776 | 3589.656 | 2018.465 | 2936.989 |  |
| ~ 1 + ln(education) + 1/age | 3781.254 | 2037.139 | 3340.551 | 998.309 | 3203.448 | 560.314 | 2813.621 | 3654.872 | 1983.323 | 3672.351 | 3601.797 | 2026.455 | 2948.999 |  |
| ~ 1 + ln(education) + 1/age + sex | 3787.215 | 2038.258 | 3346.541 | 1004.421 | 3209.186 | 565.557 | 2819.466 | 3660.5 | 1984.872 | 3678.053 | 3607.904 | 2028.182 | 2955.096 |  |
| ~ 1 + ln(education) + ln(100-age) | 3664.827 | 2001.65 | 3239.811 | 976.734 | 3075.21 | 554.161 | 2733.729 | 3639.901 | 1961.814 | 3607.514 | 3579.819 | **1996.209** | 2892.394 |  |
| ~ 1 + ln(education) + ln(100-age) + sex | 3670.879 | 2002.741 | 3245.663 | 982.842 | 3081.004 | 559.443 | 2739.396 | 3645.672 | 1963.455 | 3613.357 | 3585.93 | 1997.932 | 2898.459 |  |
| ~ 1 + 1/education | 3925.723 | 2063.751 | 3384.424 | 1000.117 | 3265.612 | 559.37 | 2844.379 | 3740.628 | 2002.788 | 3802.403 | 3698.462 | 2048.673 | 2970.006 |  |
| ~ 1 + 1/education + sex | 3931.673 | 2064.889 | 3390.492 | 1006.228 | 3271.289 | 564.656 | 2850.349 | 3746.33 | 2004.241 | 3808.162 | 3704.563 | 2050.405 | 2976.118 |  |
| ~ 1 + 1/education + age | 3694.009 | 2014.487 | 3274.291 | 987.38 | 3122.889 | 555.309 | 2762.836 | 3637.234 | 1967.555 | 3617.056 | 3577.94 | 2007.198 | 2909.974 |  |
| ~ 1 + 1/education + age + sex | 3699.997 | 2015.322 | 3280.278 | 993.484 | 3128.579 | 560.615 | 2768.687 | 3642.968 | 1968.844 | 3622.826 | 3584.053 | 2008.645 | 2916.072 |  |
| ~ 1 + 1/education + √(age) | 3711.425 | 2019.286 | 3290.002 | 990.848 | 3142.666 | 556.424 | 2774.643 | 3638.62 | 1970.302 | 3626.441 | 3580.453 | 2011.173 | 2919.229 |  |
| ~ 1 + 1/education + √(age) + sex | 3717.382 | 2020.078 | 3296.016 | 996.955 | 3148.324 | 561.716 | 2780.529 | 3644.319 | 1971.541 | 3632.17 | 3586.565 | 2012.58 | 2925.333 |  |
| ~ 1 + 1/education + age^2 | 3674.179 | 2006.947 | 3247.526 | 980.741 | 3089.526 | 553.789 | 2742.564 | 3640.462 | 1963.719 | 3610.334 | 3580.041 | 2000.893 | 2894.41 |  |
| ~ 1 + 1/education + age^2 + sex | 3680.202 | 2007.831 | 3253.466 | 986.84 | 3095.258 | 559.114 | 2748.355 | 3646.238 | 1965.072 | 3616.156 | 3586.154 | 2002.388 | 2900.497 |  |
| ~ 1 + 1/education + age^3 | 3672.277 | 2002.172 | 3229.827 | 975.233 | 3067.617 | 553.238 | 2728.386 | 3648.352 | **1961.812** | 3615.972 | 3588.204 | 1996.764 | **2884.563** |  |
| ~ 1 + 1/education + age^3 + sex | 3678.309 | 2003.049 | 3235.743 | 981.329 | 3073.358 | 558.57 | 2734.146 | 3654.138 | 1963.177 | 3621.809 | 3594.316 | 1998.26 | 2890.644 |  |
| ~ 1 + 1/education + ln(age) | 3732.957 | 2024.601 | 3306.156 | 994.185 | 3163.152 | 557.7 | 2786.834 | 3642.455 | 1973.55 | 3639.736 | 3585.709 | 2015.571 | 2928.764 |  |
| ~ 1 + 1/education + ln(age) + sex | 3738.876 | 2025.342 | 3312.194 | 1000.295 | 3168.775 | 562.977 | 2792.754 | 3648.112 | 1974.731 | 3645.414 | 3591.816 | 2016.932 | 2934.873 |  |
| ~ 1 + 1/education + 1/age | 3781.675 | 2035.698 | 3335.871 | 999.768 | 3201.262 | 560.336 | 2809.535 | 3656.757 | 1980.908 | 3673.984 | 3603.19 | 2024.795 | 2946.202 |  |
| ~ 1 + 1/education + 1/age + sex | 3787.512 | 2036.341 | 3341.944 | 1005.88 | 3206.819 | 565.587 | 2815.51 | 3662.316 | 1981.973 | 3679.555 | 3609.277 | 2026.07 | 2952.315 |  |
| ~ 1 + 1/education + ln(100-age) | 3672.427 | 2002.197 | 3239.497 | 977.39 | 3077.466 | 554.04 | 2733.449 | 3643.662 | 1961.181 | 3613.573 | 3584.203 | 1996.438 | 2891.92 |  |
| ~ 1 + 1/education + ln(100-age) + sex | 3678.426 | 2002.962 | 3245.453 | 983.491 | 3083.155 | 559.354 | 2739.254 | 3649.412 | 1962.455 | 3619.361 | 3590.316 | 1997.838 | 2898.012 |  |

*Table SM1: BIC of the models*

To determine the correction for each index, we followed a systematic procedure. First, we obtained the predicted values from the best predictor model for each index. Subsequently, these predicted values were aggregated and averaged based on the variables included in the best predictive model, that are age, education, and sex. In all cases, only age and education had an impact on the results. The BIC values of the best fitting models are written in bold font.

SM2 – Means and standard deviations of the raw scores

N = number of participants per cell; mean (standard deviation) and 95%CI.

T4

| Edu  Age | [3,5) | [5,8) | [8,13) | [13,19] |
| --- | --- | --- | --- | --- |
| [20,25) |  |  |  | N = 14; 36.93 (5.97); 29 – 45 |
| [25,30) |  |  | N = 6; 48.33 (18.74); 27 – 80 | N = 16; 40.31 (4.91); 32 – 48 |
| [30,35) |  |  | N = 8; 42.62 (8.31); 35 – 60 | N = 15; 38.8 (4.75); 33 – 50 |
| [35,40) |  |  | N = 11; 57.82 (11.47); 45 – 79 | N = 18; 48.67 (11.81); 37 – 72 |
| [40,45) | N = 1; 83 | N = 4; 52 (9.76); 39 – 60 | N = 9; 53.22 (11.04); 39 – 73 | N = 26; 45.27 (7.43); 36 – 64 |
| [45,50) |  | N = 5; 66.2 (13.14); 47 – 79 | N = 14; 54.5 (8.83); 35 – 70 | N = 16; 48.19 (11.44); 30 – 67 |
| [50,55) |  | N = 10; 66.2 (16.96); 43 – 102 | N = 11; 50.91 (12.89); 30 – 72 | N = 32; 50.84 (12.38); 34 – 80 |
| [55,60) | N = 1; 70 | N = 14; 61.64 (13.37); 45 – 90 | N = 13; 58 (12.17); 38 – 78 | N = 19; 51.79 (10.22); 34 – 71 |
| [60,65) | N = 1; 55 | N = 10; 75.1 (11.68); 55 – 90 | N = 12; 62.42 (7.45); 50 – 79 | N = 14; 62.29 (11.31); 42 – 82 |
| [65,70) | N = 3; 67.67 (9.29); 60 – 78 | N = 14; 68.79 (17.09); 43 – 104 | N = 10; 68.9 (16.41); 49 – 99 | N = 11; 69.27 (12.13); 55 – 90 |
| [70,75) | N = 4; 86 (13.66); 72 – 104 | N = 11; 92.73 (19.94); 70 – 145 | N = 12; 72.75 (15.7); 52 – 96 | N = 19; 65.53 (14.55); 49 – 95 |
| [75,80) | N = 4; 79 (6.98); 72 – 85 | N = 13; 94.08 (19.43); 55 – 129 | N = 4; 86.5 (8.58); 79 – 97 | N = 10; 68 (17.86); 44 – 110 |
| [80,85) | N = 3; 92 (6.08); 85 – 96 | N = 8; 100.5 (25.27); 55 – 129 | N = 6; 81.83 (20.26); 68 – 122 | N = 6; 79.5 (9.97); 69 – 95 |
| [85,90) | N = 3; 106.33 (34.93); 75 – 144 | N = 4; 96.5 (21.49); 70 – 122 | N = 2; 75.5 (0.71); 75 – 76 | N = 5; 94.4 (27.04); 73 – 139 |

E4

| Edu  Age | [3,5) | [5,8) | [8,13) | [13,19] |
| --- | --- | --- | --- | --- |
| [20,25) |  |  |  | N = 14; 0.14 (0.36); 0 – 1 |
| [25,30) |  |  | N = 6; 2.33 (3.67); 0 – 8 | N = 16; 0.31 (0.79); 0 – 3 |
| [30,35) |  |  | N = 8; 0.62 (1.41); 0 – 4 | N = 15; 0.6 (0.74); 0 – 2 |
| [35,40) |  |  | N = 11; 0.82 (0.87); 0 – 2 | N = 18; 0.83 (1.2); 0 – 4 |
| [40,45) | N = 1; 7 | N = 4; 2 (1.63); 0 – 4 | N = 9; 1.44 (1.59); 0 – 4 | N = 26; 0.35 (0.69); 0 – 2 |
| [45,50) |  | N = 5; 4.8 (2.28); 2 – 8 | N = 14; 1.57 (2.65); 0 – 10 | N = 16; 0.88 (1.54); 0 – 6 |
| [50,55) |  | N = 10; 2.2 (2.15); 0 – 6 | N = 11; 1.18 (2.04); 0 – 7 | N = 32; 1.66 (2.77); 0 – 14 |
| [55,60) | N = 1; 1 | N = 14; 2.29 (1.77); 0 – 6 | N = 13; 1.23 (1.64); 0 – 5 | N = 19; 0.79 (1.32); 0 – 5 |
| [60,65) | N = 1; 0 | N = 10; 2.8 (2.7); 0 – 8 | N = 12; 2.08 (2.19); 0 – 6 | N = 14; 2.07 (2.4); 0 – 9 |
| [65,70) | N = 3; 3 (3.61); 0 – 7 | N = 14; 2.93 (2.73); 0 – 7 | N = 10; 1.8 (2.3); 0 – 6 | N = 11; 2.09 (2.43); 0 – 8 |
| [70,75) | N = 4; 3.75 (0.96); 3 – 5 | N = 11; 2.45 (1.69); 0 – 5 | N = 12; 2.67 (2.61); 0 – 8 | N = 19; 1.53 (1.58); 0 – 4 |
| [75,80) | N = 4; 5 (4.08); 1 – 9 | N = 13; 2.92 (1.75); 0 – 7 | N = 4; 5 (2.94); 1 – 8 | N = 10; 2.7 (2.67); 0 – 8 |
| [80,85) | N = 3; 6.33 (3.06); 3 – 9 | N = 8; 5.25 (3.06); 0 – 9 | N = 6; 3.33 (4.46); 0 – 12 | N = 6; 2.83 (2.48); 0 – 6 |
| [85,90) | N = 3; 3 (4.36); 0 – 8 | N = 4; 8.5 (2.65); 5 – 11 | N = 2; 5 (4.24); 2 – 8 | N = 5; 5.4 (2.97); 2 – 9 |

T3

| Edu  Age | [3,5) | [5,8) | [8,13) | [13,19] |
| --- | --- | --- | --- | --- |
| [20,25) |  |  |  | N = 14; 21.29 (3.1); 17 – 27 |
| [25,30) |  |  | N = 6; 23 (5.44); 18 – 32 | N = 16; 23.44 (4.37); 17 – 32 |
| [30,35) |  |  | N = 8; 20.75 (3.37); 18 – 27 | N = 15; 21.6 (2.77); 19 – 28 |
| [35,40) |  |  | N = 11; 27.82 (5.19); 21 – 38 | N = 18; 23.78 (3.8); 20 – 33 |
| [40,45) | N = 1; 30 | N = 4; 26 (2.71); 24 – 30 | N = 9; 21.67 (3.32); 17 – 25 | N = 26; 23 (4.92); 15 – 40 |
| [45,50) |  | N = 5; 26.8 (6.98); 21 – 38 | N = 14; 26.29 (6.63); 15 – 37 | N = 16; 22.56 (5.38); 17 – 35 |
| [50,55) |  | N = 10; 30.2 (6.14); 23 – 44 | N = 11; 27 (4.36); 20 – 34 | N = 32; 22.59 (4.63); 15 – 35 |
| [55,60) | N = 1; 25 | N = 14; 28.07 (4.53); 20 – 36 | N = 13; 23.08 (5.19); 15 – 34 | N = 19; 23.11 (4.32); 18 – 32 |
| [60,65) | N = 1; 40 | N = 10; 31.7 (4.6); 25 – 37 | N = 12; 26.25 (6.27); 19 – 40 | N = 14; 24.36 (6.13); 15 – 34 |
| [65,70) | N = 3; 35.67 (4.04); 32 – 40 | N = 14; 35.43 (13.8); 21 – 64 | N = 10; 28.2 (8.05); 17 – 40 | N = 11; 30.55 (5.37); 25 – 38 |
| [70,75) | N = 4; 42.75 (21.56); 30 – 75 | N = 11; 37.82 (10.85); 27 – 60 | N = 12; 38.33 (17.9); 20 – 78 | N = 19; 27.84 (5.55); 23 – 45 |
| [75,80) | N = 4; 44.75 (7.93); 35 – 54 | N = 13; 44.85 (11.7); 32 – 68 | N = 4; 55.25 (18.08); 35 – 71 | N = 10; 34.1 (11.86); 19 – 50 |
| [80,85) | N = 3; 54.33 (11.02); 43 – 65 | N = 8; 44.62 (13.74); 27 – 65 | N = 6; 44.17 (20.27); 26 – 76 | N = 6; 32.5 (8.04); 23 – 45 |
| [85,90) | N = 3; 58 (13.11); 44 – 70 | N = 4; 55.75 (21.56); 35 – 79 | N = 2; 33 (7.07); 28 – 38 | N = 5; 37 (1.87); 34 – 39 |

E3

Effect of age:

| [20,25) | [25,30) | [30,35) | [35,40) | [40,45) | [45,50) | [50,55) | [55,60) | [60,65) | [65,70) | [70,75) | [75,80) | [80,85) | [85,90) |
| --- | --- | --- | --- | --- | --- | --- | --- | --- | --- | --- | --- | --- | --- |
| N = 14; 0.07 (0.27);  0 – 1 | N = 22; 0.14 (0.64);  0 – 3 | N = 23; 0 (0);  0 – 0 | N = 29; 0.03 (0.19);  0 – 1 | N = 40; 0.03 (0.16);  0 – 1 | N = 35; 0.03 (0.17);  0 – 1 | N = 53; 0.11 (0.61);  0 – 4 | N = 47; 0.04 (0.2); 0 – 1 | N = 37; 0.03 (0.16);  0 – 1 | N = 38; 0.16 (0.59);  0 – 3 | N = 46; 0.26 (0.68); 0 – 3 | N = 31; 0.42 (1.43); 0 – 7 | N = 23; 0.91 (1.56); 0 – 5 | N = 14; 0.79 (1.42); 0 – 5 |

T2

| Edu  Age | [3,5) | [5,8) | [8,13) | [13,19] |
| --- | --- | --- | --- | --- |
| [20,25) |  |  |  | N = 14; 24.21 (3.07); 20 – 30 |
| [25,30) |  |  | N = 6; 28.33 (8.57); 21 – 45 | N = 16; 24.81 (3.41); 19 – 31 |
| [30,35) |  |  | N = 8; 23.5 (3.85); 19 – 30 | N = 15; 23.4 (2.64); 20 – 28 |
| [35,40) |  |  | N = 11; 27.27 (4.34); 22 – 35 | N = 18; 25.67 (3.45); 21 – 32 |
| [40,45) | N = 1; 33 | N = 4; 27.5 (3.79); 25 – 33 | N = 9; 24.22 (5.24); 18 – 35 | N = 26; 24.73 (4.46); 17 – 41 |
| [45,50) |  | N = 5; 31.2 (8.98); 23 – 44 | N = 14; 27.14 (5.7); 17 – 36 | N = 16; 24.19 (4.35); 19 – 34 |
| [50,55) |  | N = 10; 32.9 (11.95); 26 – 65 | N = 11; 27.55 (3.64); 21 – 34 | N = 32; 25.94 (4.17); 19 – 39 |
| [55,60) | N = 1; 27 | N = 14; 30.93 (3.97); 26 – 39 | N = 13; 28.15 (4.47); 21 – 36 | N = 19; 26.21 (4.01); 22 – 34 |
| [60,65) | N = 1; 36 | N = 10; 33.4 (4.09); 29 – 41 | N = 12; 30 (6.22); 23 – 41 | N = 14; 27.79 (5.09); 21 – 36 |
| [65,70) | N = 3; 35.67 (2.52); 33 – 38 | N = 14; 36.07 (10.07); 24 – 52 | N = 10; 30.8 (6.16); 21 – 39 | N = 11; 32.64 (4.08); 28 – 40 |
| [70,75) | N = 4; 39.75 (8.66); 32 – 52 | N = 11; 35.09 (6.8); 27 – 47 | N = 12; 34.42 (11.19); 23 – 57 | N = 19; 31.16 (7.83); 25 – 59 |
| [75,80) | N = 4; 40.5 (7.94); 31 – 48 | N = 13; 47.54 (13.93); 29 – 73 | N = 4; 52.5 (18.19); 33 – 68 | N = 10; 32.8 (8.3); 23 – 49 |
| [80,85) | N = 3; 57.33 (14.01); 43 – 71 | N = 8; 43.5 (6.55); 36 – 56 | N = 6; 48.33 (14.76); 29 – 69 | N = 6; 39.67 (7.17); 28 – 47 |
| [85,90) | N = 3; 55.33 (5.69); 49 – 60 | N = 4; 51.25 (12.15); 36 – 62 | N = 2; 35.5 (3.54); 33 – 38 | N = 5; 43 (9.46); 34 – 59 |

E2

Effect of age:

| [20,25) | [25,30) | [30,35) | [35,40) | [40,45) | [45,50) | [50,55) | [55,60) | [60,65) | [65,70) | [70,75) | [75,80) | [80,85) | [85,90) |
| --- | --- | --- | --- | --- | --- | --- | --- | --- | --- | --- | --- | --- | --- |
| N = 14; 0.07 (0.27); 0 – 1 | N = 22; 0.14 (0.47); 0 – 2 | N = 23; 0 (0); 0 – 0 | N = 29; 0.14 (0.44); 0 – 2 | N = 40; 0 (0); 0 – 0 | N = 35; 0.03 (0.17); 0 – 1 | N = 53; 0.08 (0.27); 0 – 1 | N = 47; 0.13 (0.45); 0 – 2 | N = 37; 0.05 (0.23); 0 – 1 | N = 38; 0.24 (0.68); 0 – 3 | N = 45; 0.29 (0.73); 0 – 3 | N = 31; 0.06 (0.25); 0 – 1 | N = 23; 0.39 (0.66); 0 – 2 | N = 14; 0.21 (0.58); 0 – 2 |

T1

| Edu  Age | [3,5) | [5,8) | [8,13) | [13,19] |
| --- | --- | --- | --- | --- |
| [20,25) |  |  |  | N = 14; 22.07 (4.08); 18 – 33 |
| [25,30) |  |  | N = 6; 21.67 (3.27); 17 – 27 | N = 16; 21 (2.73); 15 – 26 |
| [30,35) |  |  | N = 8; 19.88 (3.44); 16 – 25 | N = 15; 20.6 (2.75); 17 – 28 |
| [35,40) |  |  | N = 11; 24 (2.86); 19 – 28 | N = 18; 22.17 (3.11); 18 – 28 |
| [40,45) | N = 1; 23 | N = 4; 24 (3.27); 20 – 28 | N = 9; 21.67 (3.24); 17 – 25 | N = 26; 21.88 (4.37); 16 – 38 |
| [45,50) |  | N = 5; 23.6 (7.02); 16 – 35 | N = 14; 24.07 (5.43); 15 – 34 | N = 16; 20.56 (2.71); 17 – 27 |
| [50,55) |  | N = 10; 26.8 (4.92); 22 – 36 | N = 11; 23.91 (2.84); 18 – 27 | N = 32; 21.28 (3.43); 15 – 29 |
| [55,60) | N = 1; 24 | N = 14; 24.93 (2.7); 21 – 30 | N = 13; 21.38 (3.66); 15 – 27 | N = 19; 21.58 (3.22); 18 – 28 |
| [60,65) | N = 1; 32 | N = 10; 27.9 (3.78); 22 – 33 | N = 12; 24.33 (4.01); 19 – 32 | N = 14; 23.64 (5.02); 15 – 32 |
| [65,70) | N = 3; 31 (2); 29 – 33 | N = 14; 27.64 (7.14); 18 – 39 | N = 10; 25.3 (5.52); 17 – 33 | N = 11; 25.55 (3.59); 20 – 31 |
| [70,75) | N = 4; 28.5 (4.04); 25 – 34 | N = 11; 28.45 (3.53); 23 – 34 | N = 12; 25.92 (6.44); 20 – 41 | N = 19; 23.53 (2.34); 20 – 28 |
| [75,80) | N = 4; 31.5 (5.32); 25 – 38 | N = 13; 33.62 (10.57); 24 – 66 | N = 4; 32 (6.38); 23 – 37 | N = 10; 25.7 (4.27); 19 – 32 |
| [80,85) | N = 3; 42.67 (14.57); 31 – 59 | N = 8; 33 (5.15); 27 – 41 | N = 6; 31 (6.03); 23 – 41 | N = 6; 28.17 (6.11); 18 – 35 |
| [85,90) | N = 3; 38 (6.08); 34 – 45 | N = 4; 41.25 (11.79); 27 – 55 | N = 2; 30 (5.66); 26 – 34 | N = 5; 30.4 (4.62); 24 – 36 |

T4 – T3

| Edu  Age | [3,5) | [5,8) | [8,13) | [13,19] |
| --- | --- | --- | --- | --- |
| [20,25) |  |  |  | N = 14; 15.64 (5.68); 9 – 27 |
| [25,30) |  |  | N = 6; 25.33 (14.81); 9 – 48 | N = 16; 16.88 (6.48); 6 – 31 |
| [30,35) |  |  | N = 8; 21.88 (8.89); 13 – 41 | N = 15; 17.2 (4.74); 10 – 26 |
| [35,40) |  |  | N = 11; 30 (7.86); 18 – 42 | N = 18; 24.89 (9.89); 13 – 47 |
| [40,45) | N = 1; 53 | N = 4; 26 (8.21); 15 – 34 | N = 9; 31.56 (8.71); 21 – 48 | N = 26; 22.27 (6.53); 12 – 39 |
| [45,50) |  | N = 5; 39.4 (14.67); 23 – 58 | N = 14; 28.21 (7.22); 18 – 39 | N = 16; 25.62 (10.61); 8 – 38 |
| [50,55) |  | N = 10; 36 (14.7); 19 – 67 | N = 11; 23.91 (11.51); 5 – 42 | N = 32; 28.25 (11.06); 14 – 52 |
| [55,60) | N = 1; 45 | N = 14; 33.57 (12.54); 20 – 60 | N = 13; 34.92 (10.78); 20 – 54 | N = 19; 28.68 (8.14); 14 – 41 |
| [60,65) | N = 1; 15 | N = 10; 43.4 (13.38); 19 – 62 | N = 12; 36.17 (6.28); 25 – 45 | N = 14; 37.93 (9.31); 22 – 56 |
| [65,70) | N = 3; 32 (13.11); 20 – 46 | N = 14; 33.36 (14); 10 – 60 | N = 10; 40.7 (17.18); 20 – 73 | N = 11; 38.73 (13.54); 18 – 65 |
| [70,75) | N = 4; 43.25 (24.6); 13 – 72 | N = 11; 54.91 (20.24); 17 – 95 | N = 12; 34.42 (17.96); 3 – 63 | N = 19; 37.68 (11.87); 24 – 62 |
| [75,80) | N = 4; 34.25 (9.74); 20 – 42 | N = 13; 49.23 (18.94); 10 – 81 | N = 4; 31.25 (17.33); 9 – 45 | N = 10; 33.9 (12.55); 15 – 60 |
| [80,85) | N = 3; 37.67 (5.86); 31 – 42 | N = 8; 55.88 (29.12); 16 – 102 | N = 6; 37.67 (13.53); 11 – 47 | N = 6; 47 (10.39); 34 – 65 |
| [85,90) | N = 3; 48.33 (45.37); 15 – 100 | N = 4; 40.75 (9.39); 32 – 53 | N = 2; 42.5 (6.36); 38 – 47 | N = 5; 57.4 (28.68); 35 – 105 |

E4 – E3

| Edu  Age | [3,5) | [5,8) | [8,13) | [13,19] |
| --- | --- | --- | --- | --- |
| [20,25) |  |  |  | N = 14; 0.14 (0.36); 0 – 1 |
| [25,30) |  |  | N = 6; 2.33 (3.67); 0 – 8 | N = 16; 0.31 (0.79); 0 – 3 |
| [30,35) |  |  | N = 8; 0.62 (1.41); 0 – 4 | N = 15; 0.6 (0.74); 0 – 2 |
| [35,40) |  |  | N = 11; 0.82 (0.87); 0 – 2 | N = 18; 0.78 (1.22); 0 – 4 |
| [40,45) | N = 1; 7 | N = 4; 2 (1.63); 0 – 4 | N = 9; 1.44 (1.59); 0 – 4 | N = 26; 0.35 (0.69); 0 – 2 |
| [45,50) |  | N = 5; 4.6 (1.95); 2 – 7 | N = 14; 1.57 (2.65); 0 – 10 | N = 16; 0.88 (1.54); 0 – 6 |
| [50,55) |  | N = 10; 2.2 (2.15); 0 – 6 | N = 11; 1.09 (2.07); 0 – 7 | N = 32; 1.59 (2.49); 0 – 12 |
| [55,60) | N = 1; 1 | N = 14; 2.21 (1.81); 0 – 6 | N = 13; 1.23 (1.64); 0 – 5 | N = 19; 0.79 (1.32); 0 – 5 |
| [60,65) | N = 1; 0 | N = 10; 2.8 (2.7); 0 – 8 | N = 12; 2 (2.13); 0 – 6 | N = 14; 2.07 (2.4); 0 – 9 |
| [65,70) | N = 3; 3 (3.61); 0 – 7 | N = 14; 2.71 (2.58); 0 – 6 | N = 10; 1.8 (2.3); 0 – 6 | N = 11; 2.09 (2.43); 0 – 8 |
| [70,75) | N = 4; 3.5 (1.29); 2 – 5 | N = 11; 2.18 (1.66); 0 – 5 | N = 12; 2.08 (1.73); 0 – 5 | N = 19; 1.53 (1.58); 0 – 4 |
| [75,80) | N = 4; 5 (4.08); 1 – 9 | N = 13; 2.46 (2.07); 0 – 7 | N = 4; 4.25 (2.99); 1 – 8 | N = 10; 2.7 (2.67); 0 – 8 |
| [80,85) | N = 3; 5 (3.46); 3 – 9 | N = 8; 4.38 (2.67); 0 – 8 | N = 6; 2 (2.61); 0 – 7 | N = 6; 2.5 (2.51); 0 – 6 |
| [85,90) | N = 3; 2.67 (4.62); 0 – 8 | N = 4; 7.25 (2.22); 5 – 10 | N = 2; 4 (5.66); 0 – 8 | N = 5; 4.8 (3.42); 2 – 9 |

T4 – T2

| Edu  Age | [3,5) | [5,8) | [8,13) | [13,19] |
| --- | --- | --- | --- | --- |
| [20,25) |  |  |  | N = 14; 12.71 (4.7); 5 – 21 |
| [25,30) |  |  | N = 6; 20 (11.71); 3 – 35 | N = 16; 15.5 (5.13); 8 – 24 |
| [30,35) |  |  | N = 8; 19.12 (7.92); 9 – 35 | N = 15; 15.4 (4.39); 10 – 26 |
| [35,40) |  |  | N = 11; 30.55 (9.38); 16 – 45 | N = 18; 23 (11.06); 11 – 48 |
| [40,45) | N = 1; 50 | N = 4; 24.5 (9.18); 12 – 34 | N = 9; 29 (8.67); 20 – 47 | N = 26; 20.54 (6.74); 11 – 38 |
| [45,50) |  | N = 5; 35 (9.62); 24 – 49 | N = 14; 27.36 (7.23); 18 – 41 | N = 16; 24 (10.46); 6 – 36 |
| [50,55) |  | N = 10; 33.3 (11.2); 17 – 50 | N = 11; 23.36 (11.09); 4 – 41 | N = 32; 24.91 (11.87); 9 – 51 |
| [55,60) | N = 1; 43 | N = 14; 30.71 (10.87); 18 – 58 | N = 13; 29.85 (10.16); 14 – 50 | N = 19; 25.58 (7.81); 11 – 37 |
| [60,65) | N = 1; 19 | N = 10; 41.7 (12.18); 18 – 58 | N = 12; 32.42 (5.92); 25 – 42 | N = 14; 34.5 (10.14); 20 – 52 |
| [65,70) | N = 3; 32 (11.79); 22 – 45 | N = 14; 32.71 (15.75); 11 – 61 | N = 10; 38.1 (15.24); 21 – 65 | N = 11; 36.64 (13.05); 16 – 61 |
| [70,75) | N = 4; 46.25 (13.72); 36 – 65 | N = 11; 57.64 (18.08); 37 – 100 | N = 12; 38.33 (16.58); 1 – 64 | N = 19; 34.37 (14.78); 8 – 59 |
| [75,80) | N = 4; 38.5 (1.91); 37 – 41 | N = 13; 46.54 (17.08); 11 – 79 | N = 4; 34 (17.78); 11 – 49 | N = 10; 35.2 (15.45); 16 – 69 |
| [80,85) | N = 3; 34.67 (8.74); 25 – 42 | N = 8; 57 (25.05); 19 – 91 | N = 6; 33.5 (14.87); 14 – 53 | N = 6; 39.83 (8.89); 27 – 52 |
| [85,90) | N = 3; 51 (29.82); 26 – 84 | N = 4; 45.25 (12.2); 34 – 62 | N = 2; 40 (4.24); 37 – 43 | N = 5; 51.4 (19.27); 31 – 80 |

E4 – E2

| Edu  Age | [3,5) | [5,8) | [8,13) | [13,19] |
| --- | --- | --- | --- | --- |
| [20,25) |  |  |  | N = 14; 0.07 (0.47); -1 – 1 |
| [25,30) |  |  | N = 6; 2.17 (3.82); -1 – 8 | N = 16; 0.19 (0.98); -2 – 3 |
| [30,35) |  |  | N = 8; 0.62 (1.41); 0 – 4 | N = 15; 0.6 (0.74); 0 – 2 |
| [35,40) |  |  | N = 11; 0.45 (1.13); -1 – 2 | N = 18; 0.83 (1.2); 0 – 4 |
| [40,45) | N = 1; 7 | N = 4; 2 (1.63); 0 – 4 | N = 9; 1.44 (1.59); 0 – 4 | N = 26; 0.35 (0.69); 0 – 2 |
| [45,50) |  | N = 5; 4.8 (2.28); 2 – 8 | N = 14; 1.57 (2.65); 0 – 10 | N = 16; 0.81 (1.52); 0 – 6 |
| [50,55) |  | N = 10; 2.2 (2.15); 0 – 6 | N = 11; 1.18 (2.04); 0 – 7 | N = 32; 1.53 (2.79); -1 – 14 |
| [55,60) | N = 1; 1 | N = 14; 2.07 (1.98); -1 – 6 | N = 13; 1.15 (1.68); 0 – 5 | N = 19; 0.68 (1.38); -1 – 5 |
| [60,65) | N = 1; 0 | N = 10; 2.7 (2.83); -1 – 8 | N = 12; 2.08 (2.19); 0 – 6 | N = 14; 2 (2.18); 0 – 8 |
| [65,70) | N = 3; 3 (3.61); 0 – 7 | N = 14; 2.5 (2.74); -2 – 7 | N = 10; 1.6 (2.07); 0 – 6 | N = 11; 2 (2.37); 0 – 8 |
| [70,75) | N = 4; 3.25 (0.5); 3 – 4 | N = 11; 2.45 (1.69); 0 – 5 | N = 12; 2.08 (2.27); -2 – 6 | N = 18; 1.33 (1.41); 0 – 4 |
| [75,80) | N = 4; 5 (4.08); 1 – 9 | N = 13; 2.85 (1.82); 0 – 7 | N = 4; 5 (2.94); 1 – 8 | N = 10; 2.6 (2.72); 0 – 8 |
| [80,85) | N = 3; 6.33 (3.06); 3 – 9 | N = 8; 4.75 (3.06); 0 – 9 | N = 6; 3.17 (4.62); -1 – 12 | N = 6; 2.17 (2.23); 0 – 6 |
| [85,90) | N = 3; 3 (4.36); 0 – 8 | N = 4; 8 (2.16); 5 – 10 | N = 2; 5 (4.24); 2 – 8 | N = 5; 5.2 (3.11); 2 – 9 |

T3 – T1

| Edu  Age | [3,5) | [5,8) | [8,13) | [13,19] |
| --- | --- | --- | --- | --- |
| [20,25) |  |  |  | N = 14; 0.29 (0.61); 0 – 2 |
| [25,30) |  |  | N = 6; 2.17 (2.14); 0 – 5 | N = 16; 2.69 (3.68); 0 – 13 |
| [30,35) |  |  | N = 8; 1.12 (1.55); 0 – 3 | N = 15; 1.53 (1.88); 0 – 7 |
| [35,40) |  |  | N = 11; 3.82 (2.86); 1 – 11 | N = 18; 1.83 (2.41); 0 – 8 |
| [40,45) | N = 1; 7 | N = 4; 2 (2.16); 0 – 5 | N = 9; 0.22 (0.44); 0 – 1 | N = 26; 1.31 (1.57); 0 – 7 |
| [45,50) |  | N = 5; 3.2 (3.42); 0 – 8 | N = 14; 2.36 (2.62); 0 – 8 | N = 16; 2 (3.31); 0 – 11 |
| [50,55) |  | N = 10; 3.9 (5.72); 0 – 19 | N = 11; 3.09 (2.3); 1 – 8 | N = 32; 1.69 (2.19); 0 – 10 |
| [55,60) | N = 1; 1 | N = 14; 3.21 (2.61); 0 – 9 | N = 13; 1.77 (2.28); 0 – 8 | N = 19; 1.53 (1.43); 0 – 4 |
| [60,65) | N = 1; 8 | N = 10; 3.8 (1.69); 2 – 7 | N = 12; 2 (2.92); 0 – 10 | N = 14; 1.14 (1.83); 0 – 5 |
| [65,70) | N = 3; 4.67 (3.79); 2 – 9 | N = 14; 7.79 (7.7); 1 – 25 | N = 10; 2.9 (3.03); 0 – 9 | N = 11; 5 (3.22); 1 – 10 |
| [70,75) | N = 4; 14.25 (17.88); 4 – 41 | N = 11; 9.64 (10.02); 0 – 34 | N = 12; 12.42 (13.25); 0 – 37 | N = 19; 4.32 (4.37); 0 – 17 |
| [75,80) | N = 4; 13.25 (3.2); 10 – 16 | N = 13; 11.23 (7.64); 1 – 29 | N = 4; 23.25 (12.45); 12 – 35 | N = 10; 8.4 (9.01); 0 – 22 |
| [80,85) | N = 3; 11.67 (5.51); 6 – 17 | N = 8; 11.88 (9.72); 0 – 26 | N = 6; 13.17 (14.76); 3 – 35 | N = 6; 4.67 (4.89); 0 – 14 |
| [85,90) | N = 3; 20.33 (18.18); 0 – 35 | N = 4; 14.5 (13.89); 2 – 34 | N = 2; 3 (1.41); 2 – 4 | N = 5; 6.6 (3.44); 2 – 10 |

SM3 – Percentiles distribution for T4 and E4

The following table reports the percentiles of corrected scores derived from healthy participants, together with the percentages of patients with Alzheimer’s disease and mild cognitive impairment (MCI) whose scores exceed the cut-point.

|  | **STROOP T4** | | | **STROOP E4** | | |
| --- | --- | --- | --- | --- | --- | --- |
|  | **Cut-point** | **Alzheimer** | **MCI** | **Cut-point** | **Alzheimer** | **MCI** |
| 99% | 92.94 | 58.87 | 23.02 | 8.16 | 65.32 | 29.50 |
| 98% | 88.39 | 66.13 | 27.34 | 6.87 | 69.35 | 31.65 |
| 97% | 86.21 | 69.35 | 28.78 | 6.73 | 70.16 | 32.37 |
| 96% | 82.96 | 74.19 | 34.53 | 6.25 | 70.97 | 33.81 |
| 95% | 80.58 | 77.42 | 35.25 | 5.69 | 73.39 | 36.69 |
| 94% | 78.63 | 78.23 | 39.57 | 5.41 | 74.19 | 36.69 |
| 93% | 77.65 | 78.23 | 40.29 | 5.27 | 74.19 | 38.13 |
| 92% | 75.50 | 79.84 | 42.45 | 5.09 | 74.19 | 38.85 |
| 91% | 74.88 | 80.65 | 42.45 | 4.80 | 76.61 | 41.01 |
| 90% | 74.26 | 82.26 | 45.32 | 4.45 | 77.42 | 43.17 |
| 89% | 73.41 | 85.48 | 46.04 | 4.12 | 79.03 | 46.04 |
| 88% | 72.78 | 85.48 | 47.48 | 4.00 | 79.03 | 46.04 |
| 87% | 71.95 | 85.48 | 47.48 | 3.81 | 79.03 | 48.92 |
| 86% | 70.56 | 86.29 | 52.52 | 3.74 | 79.84 | 48.92 |
| 85% | 70.14 | 86.29 | 53.96 | 3.60 | 80.65 | 50.36 |
| 84% | 69.93 | 87.10 | 53.96 | 3.45 | 80.65 | 51.80 |
| 83% | 69.19 | 88.71 | 53.96 | 3.36 | 80.65 | 52.52 |
| 82% | 68.09 | 89.52 | 59.71 | 3.32 | 80.65 | 53.24 |
| 81% | 67.62 | 89.52 | 59.71 | 3.26 | 82.26 | 53.24 |
| 80% | 66.86 | 89.52 | 59.71 | 3.19 | 83.06 | 53.96 |
| 79% | 65.58 | 90.32 | 64.03 | 2.99 | 83.87 | 56.12 |
| 78% | 65.22 | 90.32 | 64.75 | 2.92 | 83.87 | 58.27 |
| 77% | 64.44 | 91.13 | 64.75 | 2.78 | 83.87 | 59.71 |
| 76% | 63.97 | 91.13 | 66.19 | 2.45 | 85.48 | 61.87 |
| 75% | 63.57 | 91.94 | 66.91 | 2.41 | 85.48 | 61.87 |
| 74% | 63.14 | 91.94 | 66.91 | 2.27 | 85.48 | 64.75 |
| 73% | 62.57 | 91.94 | 66.91 | 2.10 | 86.29 | 66.91 |
| 72% | 62.14 | 91.94 | 68.35 | 2.06 | 86.29 | 66.91 |
| 71% | 62.00 | 91.94 | 68.35 | 2.01 | 86.29 | 67.63 |
| 70% | 61.79 | 91.94 | 69.06 | 1.99 | 86.29 | 68.35 |
| 69% | 61.35 | 91.94 | 69.78 | 1.91 | 86.29 | 69.06 |
| 68% | 60.79 | 91.94 | 69.78 | 1.81 | 86.29 | 69.78 |
| 67% | 60.32 | 92.74 | 70.50 | 1.77 | 86.29 | 70.50 |
| 66% | 60.04 | 92.74 | 70.50 | 1.74 | 86.29 | 71.94 |
| 65% | 59.34 | 92.74 | 71.22 | 1.69 | 87.10 | 71.94 |
| 64% | 58.87 | 92.74 | 71.22 | 1.57 | 87.10 | 72.66 |
| 63% | 58.64 | 92.74 | 72.66 | 1.50 | 87.90 | 72.66 |
| 62% | 58.53 | 93.55 | 73.38 | 1.41 | 88.71 | 73.38 |
| 61% | 58.28 | 94.35 | 74.10 | 1.36 | 88.71 | 74.10 |
| 60% | 58.02 | 94.35 | 74.10 | 1.35 | 88.71 | 74.10 |
| 59% | 57.69 | 94.35 | 74.82 | 1.33 | 88.71 | 74.10 |
| 58% | 57.49 | 94.35 | 74.82 | 1.31 | 88.71 | 74.10 |
| 57% | 57.39 | 94.35 | 74.82 | 1.26 | 88.71 | 76.26 |
| 56% | 57.12 | 94.35 | 75.54 | 1.20 | 88.71 | 77.70 |
| 55% | 56.81 | 94.35 | 76.26 | 1.18 | 88.71 | 77.70 |
| 54% | 56.49 | 94.35 | 76.26 | 1.16 | 88.71 | 77.70 |
| 53% | 56.24 | 94.35 | 76.26 | 1.12 | 88.71 | 77.70 |
| 52% | 55.82 | 94.35 | 77.70 | 1.11 | 88.71 | 77.70 |
| 51% | 55.44 | 95.16 | 79.14 | 1.09 | 88.71 | 77.70 |
| 50% | 55.04 | 95.16 | 79.14 | 1.06 | 88.71 | 77.70 |
| 49% | 54.64 | 95.16 | 80.58 | 1.03 | 88.71 | 77.70 |
| 48% | 54.52 | 95.16 | 81.29 | 1.01 | 88.71 | 77.70 |
| 47% | 54.14 | 95.16 | 82.73 | 0.97 | 88.71 | 78.42 |
| 46% | 53.96 | 95.16 | 83.45 | 0.94 | 88.71 | 78.42 |
| 45% | 53.48 | 95.97 | 83.45 | 0.90 | 88.71 | 79.86 |
| 44% | 53.04 | 95.97 | 84.17 | 0.86 | 88.71 | 79.86 |
| 43% | 52.76 | 95.97 | 84.89 | 0.84 | 88.71 | 79.86 |
| 42% | 52.52 | 95.97 | 84.89 | 0.81 | 88.71 | 79.86 |
| 41% | 52.36 | 95.97 | 85.61 | 0.80 | 88.71 | 79.86 |
| 40% | 52.13 | 95.97 | 86.33 | 0.77 | 88.71 | 81.29 |
| 39% | 51.86 | 95.97 | 87.05 | 0.74 | 88.71 | 81.29 |
| 38% | 51.68 | 95.97 | 87.77 | 0.69 | 88.71 | 81.29 |
| 37% | 51.35 | 95.97 | 88.49 | 0.67 | 88.71 | 81.29 |
| 36% | 50.87 | 95.97 | 89.21 | 0.64 | 88.71 | 81.29 |
| 35% | 50.56 | 96.77 | 89.21 | 0.60 | 88.71 | 82.01 |
| 34% | 50.30 | 96.77 | 89.21 | 0.57 | 88.71 | 82.01 |
| 33% | 49.89 | 96.77 | 89.21 | 0.54 | 88.71 | 82.73 |
| 32% | 49.63 | 96.77 | 89.21 | 0.52 | 88.71 | 82.73 |
| 31% | 49.42 | 98.39 | 89.21 | 0.50 | 89.52 | 83.45 |
| 30% | 49.04 | 98.39 | 90.65 | 0.46 | 89.52 | 84.17 |
| 29% | 48.85 | 99.19 | 90.65 | 0.45 | 89.52 | 84.17 |
| 28% | 48.71 | 99.19 | 90.65 | 0.44 | 89.52 | 84.17 |
| 27% | 48.49 | 99.19 | 90.65 | 0.41 | 89.52 | 84.17 |
| 26% | 48.19 | 99.19 | 90.65 | 0.40 | 89.52 | 84.17 |
| 25% | 47.64 | 99.19 | 90.65 | 0.37 | 89.52 | 84.17 |
| 24% | 47.12 | 99.19 | 90.65 | 0.35 | 89.52 | 84.17 |
| 23% | 46.86 | 99.19 | 91.37 | 0.27 | 89.52 | 85.61 |
| 22% | 46.34 | 99.19 | 91.37 | 0.23 | 89.52 | 86.33 |
| 21% | 46.21 | 99.19 | 91.37 | 0.16 | 89.52 | 87.05 |
| 20% | 45.75 | 99.19 | 92.09 | 0.08 | 89.52 | 88.49 |
| 19% | 45.13 | 99.19 | 92.81 | 0.05 | 90.32 | 88.49 |
| 18% | 44.85 | 99.19 | 92.81 | 0.00 | 91.13 | 89.21 |
| 17% | 44.69 | 99.19 | 92.81 | 0.00 | 91.13 | 89.21 |
| 16% | 44.22 | 99.19 | 92.81 | 0.00 | 91.13 | 89.21 |
| 15% | 43.70 | 99.19 | 92.81 | 0.00 | 91.13 | 89.21 |
| 14% | 43.47 | 99.19 | 92.81 | 0.00 | 91.13 | 89.21 |
| 13% | 42.90 | 99.19 | 92.81 | 0.00 | 91.13 | 89.21 |
| 12% | 42.42 | 99.19 | 93.53 | 0.00 | 91.13 | 89.21 |
| 11% | 41.96 | 99.19 | 94.24 | 0.00 | 91.13 | 89.21 |
| 10% | 40.82 | 99.19 | 94.24 | 0.00 | 91.13 | 89.21 |
| 9% | 40.07 | 99.19 | 94.24 | 0.00 | 91.13 | 89.21 |
| 8% | 39.13 | 99.19 | 94.24 | 0.00 | 91.13 | 89.21 |
| 7% | 38.68 | 99.19 | 94.96 | 0.00 | 91.13 | 89.21 |
| 6% | 37.87 | 100.00 | 95.68 | 0.00 | 91.13 | 89.21 |
| 5% | 37.02 | 100.00 | 95.68 | 0.00 | 91.13 | 89.21 |
| 4% | 36.15 | 100.00 | 95.68 | 0.00 | 91.13 | 89.21 |
| 3% | 34.82 | 100.00 | 97.12 | 0.00 | 91.13 | 89.21 |
| 2% | 32.29 | 100.00 | 97.12 | 0.00 | 91.13 | 89.21 |
| 1% | 25.83 | 100.00 | 98.56 | 0.00 | 91.13 | 89.21 |
